# Supplementary material for: The condition for dynamic stability in humans walking with feedback control
Source: PLoS Comput Biol. 2024 Mar 18;20(3):e1011861. doi: 10.1371/journal.pcbi.1011861 (PMC10997112; doi:10.1371/journal.pcbi.1011861)
Supplement: S1 Text — (PDF) [file pcbi.1011861.s001.pdf]

1 Hyperbolic solutions

We show here that the solutions provided in Equation 2 are in fact solutions of the dynamical system defined by the differential equation $\omega^2(x-p)$. We differentiate the equation for $x(t)$ by time, getting

$$\begin{aligned} & x(t) = p + (x_0 - p)\cosh(\omega t) + \frac{v_0}{\omega}\sinh(\omega t) \\ \Leftrightarrow \quad & \frac{dx(t)}{dt} = \frac{dp}{dt} + (x_0 - p)\frac{d(\cosh(\omega t))}{dt} + \frac{v_0}{\omega}\frac{d(\sinh(\omega t))}{dt} \\ \Leftrightarrow \quad & v(t) = (x_0 - p)\sinh(\omega t)\omega + v_0\cosh(\omega t). \end{aligned}$$

Deriving that by time again gives us

$$\begin{aligned} \frac{dx(t)}{dt} &= (x_0 - p)\frac{d(\sinh(\omega t)\omega)}{dt} + v_0\frac{d(\cosh(\omega t))}{dt} \\ \dot{v}(t) &= (x_0 - p)\omega^2\cosh(\omega t) + \frac{v_0}{\omega}\sinh(\omega t) \\ &= \omega^2\big((x_0 - p)\cosh(\omega t) + \frac{v_0}{\omega}\omega\sinh(\omega t) + p - p\big) \\ &= \omega^2\big((x(t) - p\big). \end{aligned}$$

□

For reference, we state here the hyperbolic identity [5]

$$c^2 - s^2 = 1 \tag{1}$$

2 Midstance-to-midstance transitions

The state at mid-stance fully determines the state for the next time interval of length T_{step}. The foot placement location is determined by Equations 3 and 4 and both halves of the the solution, from mid-stance to the step and from step to midstance, are determined by Equation 2. We use the shorthand q_n, v_n to refer to the state of these variables at midstance of the n-th step and calculate the state at the new midstance explicitly.

Intermediate Result 1. *Given a walking system parameter set and a state at midstance, q_n, v_n, the state of the system at the next midstance for progressive walking is given by*

$$\begin{pmatrix} q_{n+1} \\ v_{n+1} \end{pmatrix} = \begin{pmatrix} -cb_o + (c^2 + s^2 - cb_p)q_n - (b_d - \frac{2}{\omega}s)cv_n \\ -b_o\omega s + (2c - b_p)\omega s q_n + (c^2 + s^2 - b_d\omega s)v_n \end{pmatrix} \tag{2}$$

and for alternating walking by

$$\begin{pmatrix} x_{n+1} \\ v_{n+1} \end{pmatrix} = \begin{pmatrix} p_n + (-1)^n b_o(1-c) + (c^2 + s^2 + b_p(1-c))(x_n - p_n) + (b_d(1-c) + \frac{2}{\omega}cs)v_n \\ (-1)^{n+1}s\omega b_o - (-1)^{n+1}(2c - b_p)s\omega q_n - (b_d s\omega - c^2 - s^2)v_n \end{pmatrix}. \tag{3}$$

Proof. For a progressive walking system, given any set of control parameters b_o, b_p, b_d and a system state at midstance of the n-th step, x_n, v_n, with contact point location

p_n, we first calculate the state of the system at the time of the next foot placement, i.e. after time $t_1 = \frac{1}{2}T_{\text{step}}$, using Equation 2, as

$$\begin{aligned} \begin{pmatrix} x(t_1) \\ v(t_1) \end{pmatrix} &= \begin{pmatrix} p_n + (x_n - p_n)\cosh(\omega t_s) + \frac{v_n}{\omega}\sinh(\omega t_s) \\ (x_n - p_n)\sinh(\omega t_s)\omega + v_n\cosh(\omega t_s) \end{pmatrix} && (4) \\ &= \begin{pmatrix} p_n + (x_n - p_n)c + \frac{v_n}{\omega}s \\ (x_n - p_n)s\omega + v_n c \end{pmatrix}. && (5) \end{aligned}$$

With this, the relationships in the Intermediate Result follow from straightforward algebra. For progressive walking systems, we get

$$\begin{aligned} q_{n+1} &= x_{n+1} - p_{n+1} \\ &= p_{n+1} + (x(t_1) - p_{n+1})c + v(t_1)\frac{s}{\omega} - p_{n+1} \\ &= \big((p_n + (x_n - p_n)c + v_n\frac{s}{\omega}) - (p_n + b_o + b_p(x_n - p_n) + b_d v_n)\big)c \\ &\quad + \big((x_n - p_n)s\omega + v_n c\big)\frac{s}{\omega} \\ &= c^2 q_n + c\frac{s}{\omega}v_n - cb_o - cb_p q_n - cb_d v_n + s^2 q_n + c\frac{s}{\omega}v_n \\ &= -cb_o + (c^2 + s^2 - cb_p)q_n - (b_d - 2\frac{s}{\omega})cv_n \end{aligned} \tag{6}$$

$$\begin{aligned} v_{n+1} &= (x(t_1) - p_{n+1})s\omega + v(t_1)c \\ &= \big((p_n + (x_n - p_n)c + v_n\frac{s}{\omega}) - (p_n + b_o + b_p(x_n - p_n) + b_d v_n)\big)s\omega \\ &\quad + ((x_n - p_n)s\omega + v_n c)c \\ &= q_n cs\omega + v_n s^2 - b_o s\omega - b_p q_n s\omega - b_d v_n s\omega + q_n s\omega c + v_n c^2 \\ &= -s\omega b_o + (2c - b_p)s\omega q_n + (c^2 + s^2 - s\omega b_d)v_n \end{aligned} \tag{7}$$

For alternating walking systems, we get

$$\begin{aligned} x_{n+1} &= p_{n+1} + (x(t_1) - p_{n+1})c + v(t_1)\frac{s}{\omega} \\ &= \big(p_n + (-1)^n b_o + b_p(x_n - p_n) + b_d v_n\big) \\ &\quad + \big((p_n + (x_n - p_n)c + v_n\frac{s}{\omega}) - (p_n + (-1)^n b_o + b_p(x_n - p_n) + b_d v_n)\big)c \\ &\quad + \big((x_n - p_n)s\omega + v_n c\big)\frac{s}{\omega} \\ &= p_n + (-1)^n b_o + b_p(x_n - p_n) + b_d v_n + p_n c + (x_n - p_n)c^2 + v_n\frac{s}{\omega}c \\ &\quad - p_n c - (-1)^n b_o c - b_p(x_n - p_n)c - b_d v_n c + (x_n - p_n)s^2 + v_n c\frac{s}{\omega} \\ &= p_n + (-1)^n b_o(1 - c) + \big(c^2 + s^2 + b_p(1 - c)\big)(x_n - p_n) + \big(b_d(1 - c) + 2c\frac{s}{\omega}\big)v_n \end{aligned} \tag{8}$$

$$\begin{aligned} v_{n+1} &= (x(t_1) - p_{n+1})s\omega + v(t_1)c \\ &= \big(p_n + (x_n - p_n)c + v_n\frac{s}{\omega} - (p_n + (-1)^n b_o + b_p(x_n - p_n) + b_d v_n)\big)s\omega \\ &\quad + ((x_n - p_n)s\omega + v_n c)c \\ &= (x_n - p_n)cs\omega + v_n s^2 - (-1)^n b_o s\omega \\ &\quad - b_p(x_n - p_n)s\omega - b_d v_n s\omega + (x_n - p_n)sc\omega + v_n c^2 \\ &= -(-1)^n s\omega b_o + (2c - b_p)s\omega(x_n - p_n) + (c^2 + s^2 - b_d s\omega)v_n \end{aligned} \tag{9}$$

□

3 Derivation of Result 1

For progressive walking, let the system be on the reference state at midstance, i.e.

$$q_n = q^{\text{ref}} = 0, \qquad v_n = v^{\text{ref}} = \frac{b_o}{2\frac{s}{\omega} - b_d}. \tag{10}$$

Then we calculate the state at the next midstance with Intermediate Result 1 as

$$\begin{aligned}
q_{n+1} =& - cb_o + (c^2 + s^2 - cb_p)q_n - (b_d - \frac{2}{\omega}s)cv_n \\
=& - cb_o - (b_d - \frac{2}{\omega}s)c(\frac{b_o}{2\frac{s}{\omega} - b_d}) \\
=& - cb_o + cb_o \\
=& \ 0 \\
=& \ q_n
\end{aligned}$$

$$\begin{aligned}
v_{n+1} =& - b_o\omega s + (2c - b_p)\omega s q_n + (c^2 + s^2 - b_d\omega s)v_n \\
=& - b_o\omega s + (c^2 + s^2 - b_d\omega s)(\frac{b_o}{2\frac{s}{\omega} - b_d}) \\
=& \frac{(-b_o\omega s)(2\frac{s}{\omega} - b_d)}{2\frac{s}{\omega} - b_d} + \frac{(2s^2 + 1 - b_d\omega s)b_o}{2\frac{s}{\omega} - b_d} \\
=& \frac{-b_o\omega s 2\frac{s}{\omega} + b_o\omega s b_d + 2s^2 b_o + b_o - b_d\omega s b_o}{2\frac{s}{\omega} - b_d} \\
=& \frac{-2b_o s^2 + 2s^2 b_o + b_o}{2\frac{s}{\omega} - b_d} \\
=& \frac{b_o}{2\frac{s}{\omega} - b_d} \\
=& \ v_n
\end{aligned}$$

For alternate walking, let the system be on the reference state at midstance, i.e.

$$q_n = x_n - p_n = q_n^{\text{ref}} = (-1)^n \frac{b_o}{2c - b_p}, \qquad v_n = v^{\text{ref}} = 0, \tag{11}$$

and we calculate the state at the next midstance as

$$
\begin{aligned}
x_{n+1} &= p_n + (-1)^n b_o(1-c) + (c^2 + s^2 + b_p(1-c))(x_n - p_n) \\
&\quad + (b_d(1-c) + \frac{2}{\omega}cs)v_n \\
&= p_n + (-1)^n b_o - (-1)^n b_o c + b_p(x_n - p_n) \\
&\quad + (c^2 + s^2 - b_p c)(x_n - p_n) \\
&= p_n + (-1)^n b_o + b_p(x_n - p_n) - (-1)^n b_o c \\
&\quad + (2c^2 + 1 - b_p c)(-1)^n \frac{b_o}{2c - b_p} \\
&= p_{n+1} - (-1)^n b_o c + (-1)^n \frac{b_o(2c^2 + 1 - b_p c)}{2c - b_p} \\
&= p_{n+1} - (-1)^n \frac{b_o c(2c - b_p)}{2c - b_p} + (-1)^n \frac{b_o(2c^2 + 1 - b_p c)}{2c - b_p} \\
&= p_{n+1} - (-1)^n \frac{b_o(2c^2 - b_p c) - b_o(2c^2 + 1 - b_p c)}{2c - b_p} \\
&= p_{n+1} - (-1)^n \frac{-b_o}{2c - b_p} \\
&= p_{n+1} - q_n \\
\Leftrightarrow \qquad q_{n+1} &= -q_n
\end{aligned}
$$

$$
\begin{aligned}
v_{n+1} =& (-1)^{n+1} s\omega b_o - (-1)^{n+1}(2c - b_p)s\omega q_n - (b_d s\omega - c^2 - s^2)v_n \\
=& (-1)^{n+1} s\omega b_o - (-1)^{n+1}(2c - b_p)s\omega(-1)^n \frac{b_o}{2c - b_p} \\
=& (-1)^{n+1} s\omega b_o - (-1)^{n+1}(2c - b_p)s\omega(-1)^n \frac{b_o}{2c - b_p} \\
=& \ 0 \\
=& \ v_n
\end{aligned}
$$

This implies that $q_{n+2} = -q_{n+1} = q_n$, and $v_{n+2} = v_n$ □

4 Derivation of Result 2

We prove this by calculating δ_{n+1} explicitly. For progressive walking systems, we get

$$
\begin{aligned}
\delta^{(q)}_{n+1} &= q_{n+1} - q^{\text{ref}} \\
&= -cb_o + (c^2 + s^2 - cb_p)q_n - (b_d - 2\frac{s}{\omega})cv_n \\
&= -cb_o + (c^2 + s^2 - cb_p)(\delta^{(q)}_n + q^{\text{ref}}) - (b_d - 2\frac{s}{\omega})c(\delta^{(v)}_n + v^{\text{ref}}) \\
&= -cb_o + (c^2 + s^2 - cb_p)\delta^{(q)}_n - (b_d - 2\frac{s}{\omega})c(\delta^{(v)}_n + \frac{b_o}{2\frac{s}{\omega} - b_d}) \\
&= -cb_o + (c^2 + s^2 - cb_p)\delta^{(q)}_n - (b_d - 2\frac{s}{\omega})c\delta^{(v)}_n - (b_d - 2\frac{s}{\omega})c\frac{b_o}{2\frac{s}{\omega} - b_d}) \\
&= (c^2 + s^2 - cb_p)\delta^{(q)}_n + (2\frac{s}{\omega} - b_d)c\delta^{(v)}_n
\end{aligned}
$$

$$
\begin{aligned}
\delta_{n+1}^{(v)} &= v_{n+1} - v^{\text{ref}} \\
&= -s\omega b_o + (2c - b_p)s\omega q_n + (c^2 + s^2 - s\omega b_d)v_n - \frac{b_o}{2\frac{s}{\omega} - b_d} \\
&= -s\omega b_o + (2c - b_p)s\omega(\delta_n^{(q)} + q^{\text{ref}}) + (c^2 + s^2 - s\omega b_d)(\delta_n^{(v)} + v^{\text{ref}}) - \frac{b_o}{2\frac{s}{\omega} - b_d} \\
&= -s\omega b_o + (2c - b_p)s\omega\delta_n^{(q)} + (c^2 + s^2 - s\omega b_d)(\delta_n^{(v)} + \frac{b_o}{(2\frac{s}{\omega} - b_d))} - \frac{b_o}{2\frac{s}{\omega} - b_d} \\
&= -s\omega b_o + (2c - b_p)s\omega\delta_n^{(q)} + (c^2 + s^2 - s\omega b_d)\delta_n^{(v)} \\
&\quad + (c^2 + s^2 - s\omega b_d)\frac{b_o}{2\frac{s}{\omega} - b_d} - \frac{b_o}{2\frac{s}{\omega} - b_d} \\
&= -s\omega b_o + (2c - b_p)s\omega\delta_n^{(q)} + (c^2 + s^2 - s\omega b_d)\delta_n^{(v)} \\
&\quad + (2s^2 + 1 - s\omega b_d)\frac{b_o}{2\frac{s}{\omega} - b_d} - \frac{b_o}{2\frac{s}{\omega} - b_d} \\
&= (2c - b_p)s\omega\delta_n^{(q)} + (c^2 + s^2 - s\omega b_d)\delta_n^{(v)}
\end{aligned}
$$

For alternating walking systems, we get

$$
\begin{aligned}
\delta_{n+1}^{(q)} &= q_{n+1} - q_{n+1}^{\text{ref}} \\
&= x_{n+1} - p_{n+1} - (-1)^{n+1}\frac{b_o}{2c - b_p} \\
&= p_n + (-1)^n b_o(1 - c) + (c^2 + s^2 + b_p(1 - c))(x_n - p_n) + (b_d(1 - c) + 2c\frac{s}{\omega})v_n \\
&\quad - (p_n + (-1)^n b_o + b_p(x_n - p_n) + b_d v_n) - (-1)^{n+1}\frac{b_o}{2c - b_p} \\
&= -(-1)^n b_o c + (c^2 + s^2 - b_p c)q_n + (2\frac{s}{\omega} - b_d)c v_n - (-1)^{n+1}\frac{b_o}{2c - b_p} \\
&= -(-1)^n b_o c + (c^2 + s^2 - b_p c)(\delta_n^{(q)} + q_n^{\text{ref}}) \\
&\quad + (2\frac{s}{\omega} - b_d)c(\delta_n^{(v)} + v^{\text{ref}}) - (-1)^{n+1}\frac{b_o}{2c - b_p} \\
&= -(-1)^n b_o c + (c^2 + s^2 - b_p c)(\delta_n^{(q)} + (-1)^n\frac{b_o}{2c - b_p}) \\
&\quad + (2\frac{s}{\omega} - b_d)c\delta_n^{(v)} - (-1)^{n+1}\frac{b_o}{2c - b_p} \\
&= -(-1)^n\frac{(b_o c(2c - b_p) - b_o - (c^2 + s^2 - b_p c)b_o)}{2c - b_p} \\
&\quad + (c^2 + s^2 - b_p c)\delta_n^{(q)} + (2\frac{s}{\omega} - b_d)c\delta_n^{(v)} \\
&= -(-1)^n\frac{(b_o c(2c - b_p) - b_o - (2c^2 - 1 - b_p c)b_o)}{2c - b_p} \\
&\quad + (c^2 + s^2 - b_p c)\delta_n^{(q)} + (2\frac{s}{\omega} - b_d)c\delta_n^{(v)} \\
&= (c^2 + s^2 - c b_p)\delta_n^{(q)} - (b_d - 2\frac{s}{\omega})c\delta_n^{(v)};
\end{aligned}
$$

$$
\begin{aligned}
\delta_{n+1}^{(v)} &= v_{n+1} - v^{\mathrm{ref}} \\
&= -(-1)^n b_o s\omega + (2c - b_p)s\omega q_n + (c^2 + s^2 - b_d s\omega)v_n \\
&= -(-1)^n b_o s\omega + (2c - b_p)s\omega(\delta_n^{(q)} + q_n^{\mathrm{ref}}) + (c^2 + s^2 - b_d s\omega)(\delta_n^{(v)} + v^{\mathrm{ref}}) \\
&= -(-1)^n b_o s\omega + (2c - b_p)s\omega(\delta_n^{(q)} + (-1)^n \frac{b_o}{2c - b_p}) + (c^2 + s^2 - b_d s\omega)\delta_n^{(v)} \\
&= -(-1)^n (b_o s\omega - (2c - b_p)s\omega \frac{b_o}{2c - b_p}) + (2c - b_p)s\omega\delta_n^{(q)} + (c^2 + s^2 - b_d s\omega)\delta_n^{(v)} \\
&= (2c - b_p)s\omega\delta_n^{(q)} + (c^2 + s^2 - s\omega b_d)\delta_n^{(v)}
\end{aligned}
$$

This proves that $\delta_{n+1} = A\delta_n$. We can iterate this process to calculate how a deviation δ_0 from the periodic orbit at midstance propagates over k steps as

$$
\delta_k = A\delta_{k-1} = A^k \delta_0 \tag{12}
$$

From linear algebra, we know that $\lim \delta_k = 0$ if and only if the spectral radius $\rho(A)$, i.e. the largest absolute Eigenvalue of A, is less than 1 [6]. □

5 Derivation of Result 3

We first calculate the determinant of A as

$$
\begin{aligned}
\det(A) &= (c^2 + s^2 - cb_p)(c^2 + s^2 - s\omega b_d) - (2\frac{s}{\omega} - b_d)c(2c - b_p)s\omega \\
&= c^4 + 2c^2s^2 - b_p c^3 + s^4 - cs^2 b_p - c^2 s\omega b_d - s^3\omega b_d + cs\omega b_p b_d \\
&\quad - 4c^2s^2 + 2c^2 s\omega b_d + 2cs^2 b_p - cs\omega b_d b_p \\
&= (c^2 - s^2)^2 - (c^2 - s^2)cb_p + (c^2 - s^2)s\omega b_d \\
&= 1 - 1cb_p + 1s\omega b_d
\end{aligned}
$$

The Eigenvalues of the square matrix A are then calculated as

$$
\lambda_{1,2} = \frac{\mathrm{tr}(A) \pm \sqrt{D}}{2}, \tag{13}
$$

with trace

$$
\begin{aligned}
\mathrm{tr}(A) &= (-cb_p + c^2 + s^2) + (-b_d\omega s + c^2 + s^2) \\
&= -cb_p + c^2 + s^2 - b_d\omega s + c^2 + s^2 \\
&= 2(c^2 + s^2) - (cb_p + \omega s b_d)
\end{aligned}
$$

and discriminant

$$
\begin{aligned}
D = {} & \operatorname{tr}(A)^2 - 4\det(A) \\
&= (2(c^2+s^2) - (cb_p + s\omega b_d))^2 - 4(1 - cb_p + s\omega b_d) \\
&= (2c^2 + 2s^2 - cb_p - s\omega b_d)(2c^2 + 2s^2 - cb_p - s\omega b_d) - 4 + 4cb_p - 4s\omega b_d \\
&= 4c^4 + 4s^2c^2 - 2c^3b_p - 2c^2 s\omega b_d + 4c^2s^2 + 4s^4 - 2cs^2b_p - 2s^3\omega b_d \\
&\qquad - 2c^3b_p - 2cs^2b_p + c^2b_p^2 + cs\omega b_p b_d - 2c^2 s\omega b_d - 2s^3\omega b_d + cs\omega b_p b_d \\
&\qquad + s^2\omega^2 b_d^2 - 4 + 4cb_p - 4s\omega b_d \\
&= 4(c^2+s^2)^2 - 4(c^2+s^2-1)cb_p - 4(c^2+s^2+1)s\omega b_d \\
&\quad + c^2b_p^2 + 2cs\omega b_p b_d + s^2\omega^2 b_d^2 - 4 \\
&= 4(c^2+s^2)^2 - 4(2s^2+1-1)cb_p - 4(2c^2-1+1)s\omega b_d \\
&\quad + c^2b_p^2 + 2cs\omega b_p b_d + s^2\omega^2 b_d^2 - 4 \\
&= 4(c^2+s^2)^2 - 8s^2cb_p - 8c^2s\omega b_d + (cb_p + s\omega b_d)^2 - 4
\end{aligned}
$$

From Result 2 we know that a given walking system is stable if and only if the largest absolute Eigenvalue of A is smaller than 1. We calculate limits of the region of parameter sets (b_p, b_v) for which this is true, i.e. the pairs (b_p, b_v) for which A has an Eigenvalue that is exactly 1.

We will first consider the case $D >= 0$. In this case, we get

$$
\begin{aligned}
& & |\lambda_{1,2}| = \left|\frac{\operatorname{tr}(A) \pm \sqrt{D}}{2}\right| &\overset{!}{=} 1 \\
&\Leftrightarrow & \left(\operatorname{tr}(A) \pm \sqrt{D}\right)^2 &= 4 \\
&\Leftrightarrow & \operatorname{tr}(A)^2 \pm 2\operatorname{tr}(A)\sqrt{D} + D &= 4 \\
&\Leftrightarrow & \operatorname{tr}(A)^2 + D - 4 &= \mp 2\operatorname{tr}(A)\sqrt{D} \\
&\Leftrightarrow & \operatorname{tr}(A)^4 + 2\operatorname{tr}(A)^2(D-4) + (D-4)^2 &= 4\operatorname{tr}(A)^2 D \\
&\Leftrightarrow & \operatorname{tr}(A)^4 - 2\operatorname{tr}(A)^2 D - 8\operatorname{tr}(A)^2 + (D-4)^2 &= 0 \\
&\overset{(*)}{\Leftrightarrow} & (2s - b_d\omega)(b_p - 2c) &= 0 \\
&\Leftrightarrow & b_p = 2c \qquad \text{or} \qquad b_d = 2\frac{s}{\omega}
\end{aligned}
$$

Here the equivalency marked with $(*)$ can be shown by straightforward, but very lengthy, algebra. Instead of reproducing the equations here, we refer the reader to the code in the supplementary materials where this derivation is performed using the symbolic math toolbox in MATLAB.

If the discriminant is negative, the two Eigenvalues are a complex conjugate pair with

$$
\lambda_{1,2} = \frac{1}{2}\operatorname{tr}(A) \pm i\frac{1}{2}\sqrt{-D}. \tag{14}
$$

With this, we get

$$
\begin{aligned}
& & |\lambda_1| &= 1 \\
&\Leftrightarrow & |\lambda_1|^2 &= 1 \\
&\Leftrightarrow & \lambda_1\overline{\lambda}_1 &= 1 \\
&\Leftrightarrow & \frac{1}{4}\operatorname{tr}(A)^2 + \frac{1}{4}(-D) &= 1 \\
&\overset{(**)}{\Leftrightarrow} & b_p c &= b_d \omega s
\end{aligned}
$$

where again the equivalency marked with $(**)$ can be shown by straightforward, but very lengthy, algebra and is provided in the supplementary materials.

We have shown that the set of points where the absolute value of either Eigenvalue of A is given by three lines in the parameter plane. Since the function mapping a matrix to its Eigenvalues is continuous, the stability of the walking system can only change when crossing any of these three lines, because the largest absolute Eigenvalue cannot switch between being greater and lesser than 1 without becoming 1, i.e. crossing one of the three lines above.

These three lines partition the parameter plane in seven regions (see Fig 5), and all points within any one of these seven regions are either stable or unstable. To determine the stability of each of these regions, it is thus sufficient to determine it for any one single point in that region. We have performed these calculations with the symbolic toolbox in MATLAB, with the code provided in the supplementary materials. Note that we only fixed representative values for the two parameters spanning the plane, b_p and b_d. We left the other two parameters affecting stability, ω and T_{step}, free and solved the resulting equations symbolically. The results of these calculations show that the largest absolute Eigenvalue is < 1 only in the triangular region satisfying the three three inequalities in Equation 13, completing the proof. □

6 Parameter estimates

Here we show that the slope parameters of the linear regression following [1] are estimates of the control gains. Replacing notation with the one used here, using CoM instead of pelvis, and midstance, the linear regression is used to fit the Jacobian J in the equation

$$\Delta p_{n+1} = J \begin{pmatrix} \Delta x_n \\ \Delta v_n \end{pmatrix}, \tag{15}$$

where

$$J = \begin{pmatrix} \frac{\partial p_{n+1}}{\partial x_n} & \frac{\partial p_{n+1}}{\partial v_n} \end{pmatrix} = \begin{pmatrix} \frac{\partial(p_n + b_o + b_p(x_n - p_n) + b_d v_n)}{\partial x_n} & \frac{\partial(p_n + b_o + b_p(x_n - p_n) + b_d v_n)}{\partial v_n} \end{pmatrix} = \begin{pmatrix} b_p & b_d \end{pmatrix} \tag{16}$$

This shows that the slope parameters of the linear regression for CoM position, β_p, and velocity, β_d, are estimates of the control gains b_p and b_d. We calculated these slopes relating the variations in mediolateral center of mass position and velocity during midstance to variations in mediolateral foot placement (at heelstrike), as described in many of our previously published papers [2–4]. The goodness of fit was assessed via the R^2, and typically exceeded 0.7.

References

1. Wang Y, Srinivasan M. Stepping in the direction of the fall: the next foot placement can be predicted from current upper body state in steady-state walking. Biology Letters. 2014;10:20140405.

2. van Leeuwen AM, van Dieën JH, Daffertshofer A, Bruijn SM. Ankle muscles drive mediolateral center of pressure control to ensure stable steady state gait. Scientific Reports. 2021;11(1):21481. doi:10.1038/s41598-021-00463-8.

3. Van Leeuwen AM, Van Dieën JH, Bruijn SM. The effect of external lateral stabilization on ankle moment control during steady-state walking. Journal of Biomechanics. 2022;142:111259. doi:10.1016/j.jbiomech.2022.111259.

4. Fettrow T, Reimann H, Grenet D, Thompson E, Crenshaw J, Higginson J, et al. Interdependence of balance mechanisms during bipedal locomotion. PLoS ONE. 2019;14(12):15.

5. Spiegel MR, Lipschutz S, Liu J. Mathematical handbook of formulas and tables. 3rd ed. Schaum's outline series. New York: McGraw-Hill; 2009.

6. Shores TS. Applied Linear Algebra and Matrix Analysis. Undergraduate Texts in Mathematics. Cham: Springer International Publishing; 2018. Available from: `http://link.springer.com/10.1007/978-3-319-74748-4`.
