# Supplementary material for: The condition for dynamic stability in humans walking with feedback control
Source: PLoS Comput Biol. 2024 Mar 18;20(3):e1011861. doi: 10.1371/journal.pcbi.1011861 (PMC10997112; doi:10.1371/journal.pcbi.1011861)
Supplement: Attachment — Submitted filename: PCOMPBIOL-D-23-00629-review.pdf [file pcbi.1011861.s002.pdf]

Review: *PLOS Computational Biology*, PCOMPBIOL-D-23-00629.

Title: The condition for dynamic stability in humans ...

Authors: H. Reimann and S. Bruijn

This paper is centered on the mathematical analysis of the local stability of periodic steady states of the well-known linearized inverted pendulum (LIP) model of human walking. A closed loop version of the LIP with position plus velocity feedback is the focus of the study, which also includes a comparison with experimental data. Such mathematical studies of the LIP and similar dynamic walking models are worthwhile and, in my opinion, should be done more frequently, as they have the potential to generate new insights into walking dynamics and issues of clinical interest, in particular the factors underlying fall risk and recovery. At the same time, mathematical analyses of walking models, when carefully compared to experimental data, can help us understand the limitations of individual models.

However, while this general aim of the paper is worthwhile and the core results are novel and have some merit, the manuscript suffers from an imprecise and, frankly, at times sloppy presentation of the material, inadequate engagement with the literature, and results that are not particularly coherent with the aims suggested by the introduction. Thus, I cannot recommend publication of the paper, at least not without a substantial rewrite and restructuring.

General Comments

1. The main results are presented in three "theorems". This is an inappropriate name for what are straightforward applications of standard local stability theory. In general, the word "theorem" is reserved for results of considerable importance and generality. The authors are, in fact, using a theorem to analyze stability of the fixed point of a map: namely, that all eigenvalues λ of the Jacobian of the map evaluated at the fixed point must have $|\lambda| \leq 1$ for stability, and $|\lambda| < 1$ for asymptotic stability. See, for example, Theorem 1.13 in Seydel (2010), section 1.4. Nor are these results "lemmas", which are results of lesser importance needed in the proof of a theorem. That said, I understand that the authors may want to label the results so that they can be referred to them later: I suggest simply calling them "Result 1", "Result 2",..., etc.

2. Likewise, it is somewhat overblown to label as "proofs" the calculations needed to find the fixed points (periodic orbits) and assess their stability. People who regularly do such work in dynamics and control would most likely simply say they were "calculating", "deriving", or "demonstrating" the results (or some similar language). Rather than labeling them as proofs in the appendices, they should just say "Derivation of Result 1", etc.

3. Finally, while it is appropriate to display the main steps needed for these deriva-

tions in the appendices, showing so many steps of what are routine algebraic manipulations detracts from the paper. The inelegant mathematical presentation obscures the underlying simplicity of the analysis: those who already understand the basic theory find such a presentation tedious, whereas those who don't understand are not likely to find the many lines of algebra all that instructive.

4. Please note that in the above comments I am not criticizing the results, only their presentation. (In fact, I confirmed the accuracy of the calculations myself.) The point here isn't pedantry, but to achieve clarity and to accurately represent the level of the authors' achievement.

5. The strongest aspect of the paper is the stability analysis itself, culminating in the identification of the triangular stability region in the control parameter space (Fig. 4) and its dependency on cadence (Fig. 5). As summarized by the statement in lines 381–385:

   > These two examples indicate that the region of stable parameter sets shifts towards higher control gains for slower-paced walking. This means that when walking at a slower cadence, with longer step times, control gains need to be higher, or the system will not be stable. Similarly, when walking at faster stepping cadences, with shorter step times, control gains need to be lower.

   This is indeed an interesting observation! Unfortunately, the paper's pivot to testing against data seems to have distracted the authors from a more careful discussion in purely analytical terms. How precisely does their mathematical analysis compare/contrast with similar work (of Hurmuzlu, Hof, Ruina, Srinivasan, Kuo, Dingwell, to name a few just off the top of my head)? This is especially unfortunate since the second author is quite knowledgeable about such matters, having carried out a systematic review of gait stability analysis. How does this analytical work inform, clarify, correct, or move beyond previous such efforts?

6. The authors provide a partial response to the above question by pointing out that previous model-based efforts haven't tended to integrate biomechanical and neuromotor aspects of locomotion: this is largely true and an important observation. However, they neglect to note that exactly this perspective was taken in Patil et al. (2020, 2022), in which motor regulation and mechanical models were integrated to study not just local stability, but global stability and the even more general concept of viability introduced in Zaytsev et al. (2018); Zaytsev and Ruina (2003). In fact, whereas Patil et al. focus on a walker with swing leg dynamics, the work of Zaystev et al. includes a sophisticated stability analysis of the LIP that goes well beyond what's presented in this paper. Such work must be acknowledged and the relationship of this study to it and similar efforts should be discussed, particularly regarding what is novel about the authors' analysis.

7. Returning to the comparison with experimental data: it's clear that the match

isn't as good as one would hope, but I'd argue that it might be better than the authors realize. Their speculations on the sources of discrepancies focus on how:

(a) the model does not account for energy loss from collisions at heel strike;

(b) their analysis used a uniform effective leg length across all subjects; and

(c) the model does not incude the effect of ankle roll.

Of these, (b) is the most immediately relevant because it requires no change to the LIP model. Surely, including the correct leg length for each participant's data can and should be done. In contrast, as they point out, (a) would require a change in the model itself, at least during "heel strike". However, I don't believe the conservative nature of the model would have a large impact on this study: at steady state, the energy is in balance (as the authors point out), so *near* the steady state it will be *close* to being in balance and, thus, it shouldn't have a large impact on a local stability analysis. The issue of ankle roll, (c), would require a change in the model during the continuous-time swing phase. To their credit, they do check its importance by looking at data from trials with restricted ankle roll (Fig. 6), but they fail to show how this restricted data changes the plots of Fig. 5, which should be done. Furthermore, they don't say how the model would be changed to include this effect: would it be purely kinematic, or would some form of actuation, such as an ankle torque, be needed?

8. Beyond these sources of discrepancy, however, the authors miss several that are, in my opinion, likely to be as, or more, relevant:

   (a) First and foremost, one should consider parameter estimation errors. The linear regressions used to estimate the Jacobians are not discussed in sufficient detail, particularly regarding their goodness of fit. It appears from Fig. 5A that the estimates of b_p are reasonably good and are consistent with the hypothesis of cadence dependence (Fig. 5B). In contrast, the model consistently over-predicts the value of b_d obtained from the data. When models disagree with data, the problem could be the model, it could be the data quality (it might be too imprecise or noisy, for example), it could be the data analysis, or it could be some combination of all three. One does not know *a priori* where the problem lies. The authors need to consider sources of error that lie entirely in the nature of the data and its processing.

   (b) Second, the controller might be wrong. The fact that b_d is a poor fit might indicate that velocity feedback isn't being used (or is only weakly used). Indeed, if one sets $b_d = 0$, the controller is the same as Hof's "constant offset" model with position feedback, described in his 2008 paper cited by the authors. Perhaps it was a mistake to include velocity feedback in the first place?

   (c) Third, the model uses a constant value of the the parameter p (described as "the contact point between the leg and the ground", line 121). Could it be that this needs to be taken as time dependent during each step?

9. Finally, the authors spend a significant portion of the introduction talking about Hof's XCoM and MoS concepts, stating: "We postulate that the main reason that the MoS does not work well as a stability measure in walking is that it neglects the control aspect." But, while the Discussion does again mention MoS, the authors never return to examine their postulate. Does their work in some way resolve the "paradoxes" they refer to? If that isn't an aim of the paper, why focus on it so much in the introduction?

Specific Comments

1. **Lines 48–52:** Other than that the authors are making a distinction between standing and walking, I don't understand this passage, especially the part about how "the qualifier is not useful". What qualifier?

2. **Fig. 1 and lines 200–206:** The authors say the red and yellow solutions are the same as the blue solution. They certainly don't look the same!

3. **Fig. 2, Theorem 1, and supporting discussion throughout:** Like Hof (and others), the authors treat the frontal and sagittal directions as uncoupled and independent LIPs. I also understand why the authors have chosen to use x and v for position and velocity in both directions, namely, to keep the notation simple. However, the downside of this is the possibility of conceptual confusion. Specifically, one must realize that Eqs. (3) and (4) are not part of two separate systems. The lateral dynamics can't have different periodic orbits than the sagittal dynamics: they're both happening together in the same 4D phase space with states that might be written as (x_A, v_A, x_P, v_P), where subscripts A and P refer to the "alternating" and "progressive" components of the system, respectively. So, in particular, Theorem 1 is misleading if not interpreted carefully: the overall periodic gait must have period $2T_{\text{step}}$, even though the forward ("progressive") direction repeats every T_{step} time units.

4. Continuing with the above comment, the control gains b_p and b_d were selected to be the same for both directions in simulations (only the offset b_0 is different). There's no problem with this if one merely wants to study qualitatively reasonable-looking CoM trajectories, but there's no *a priori* reason to think they would be the same in a human. In this analysis, motion in the two directions (front to back or "progressive" vs. side to side or "alternating") will both be stable or unstable *at the same time*. But why should we assume that's the case? Moreover, what impact would such a difference have in Figs. 4 and 5?

5. **Line 266:** "Note that this limit line is only a choice of the illustration." I don't understand what this means. What choice is being made?

6. **Line 277:** The line stops in the middle of a sentence.

7. **Section 2.3 Stability:** The authors need to make it clear that they're focusing

strictly on *local* or *linear* stability, which deals with how the system responds to *sufficiently small* perturbations of its state. As pointed out in General Comment **6**, there are other, more general notions of stability (global stability, viability, possibly others) being examined by researchers studying bipedal walking. The authors don't have to do anything with these other definitions, but they do need to state that they're looking only at local stability. There's nothing wrong with doing that, but clarity is required. Likewise, they should include this as a significant limitation in the discussion starting on line 591.

8. **Line 290–291:** "As implied by Theorem 1, each walking system has exactly one periodic orbit." The way it is constructed, this is not what Theorem 1 does. It finds *a* periodic orbit. It does not show that that is the *only* periodic orbit.

9. **Theorem 2:** This is not quite correct. The system is stable when the eigenvalues of A are ≤ 1; it's *asymptotically* stable when they're strictly < 1. Thus, the transition case occurs when they equal 1 exactly.

10. **Fig. 4 and related discussion:** First, the word "eigenvalue" should not be capitalized if it's not at the beginning of a sentence. Second, at first glance, from Fig. 4 one gets the impression that the authors are using ρ to be an eigenvalue of A. However, they don't at this point define the symbol, so we have to guess at its meaning. It's not until 13 pages later that they say that $\rho(A)$ is the spectral radius, which is the largest absolute value (or *magnitude*) of the eigenvalues. Eigenvalues and spectral radius are related, but not the same thing. One might suspect that the authors have confused the two. A better (and conventional) way to to present this is to say that the eigenvalues of A are λ_1 and λ_2, and $\rho = \max(|\lambda_1|, |\lambda_2|)$ is the spectral radius. This should be stated clearly in or before Theorem 2 and in Fig. 4's caption.

11. **Line 356:** Same problem as above. "Color represents the largest absolute Eigenvalue $\rho(A)$" should be rewritten as "Color represents the value of the spectral radius $\rho(A)$, which is the largest absolute value of the eigenvalues of A."

12. **Section 3:** It makes more sense to say "Predictions and Results", since predictions come before the results.

13. **Fig. 5:** The estimated parameter values results are presented, and for details the reader is referred to Appendix 6.6. However, that appendix just gives the calculus for the Jacobian. It doesn't say how the data was processed to perform the regression estimates, nor does it talk about how their quality was assessed (i.e., goodness of fit estimates).

14. Continuing from the above comment, it's important to explain how the data in the two movement directions were processed. Where both sagittal plane and frontal plan components merged to estimate one pair (b_p, b_d)? Or were the gains estimated independently in both directions? This is yet another possible source of observed discrepancies between model and data.

15. **Lines 406–408:** here and throughout, the estimated values of b_p and b_d are referred to as "slopes". It would be more clear to simply call them parameter estimates. A standard notation in such cases is to say $\widehat{b}_p$ is the estimated value of b_p, etc. Introducing new regression parameters β_p and β_d (what the authors call the "slopes") and then saying these are, in fact, equal to the estimated values of b_p and b_d is completely unecessary.

16. **Lines 429–430 and 525:** "Overall, this shows that the model predicts some aspects of human stability control with foot placement ..." *What* aspects? This is never made clear!

17. **Line 466–474:** This passage is confusingly written and, frankly, wrong. *Obviously* people take steps when walking. The MoS *does not* specify "how close the biomechanical state of the system is to the threshold of having to take a step" because people are continuously "having to take steps" when walking! The MoS specifies when people would have to take specific actions to avoid falling *while* taking steps during steady gait (such as crossover steps, or shifting their CoM with their torso and arms, etc.).

18. **Lines 475–478:** This is the first place the authors mention asymptotic stability. It should be mentioned much earlier, certainly before/while presenting the methods. And what on Earth is "mathematical extremism"? I'd say the theory of stability isn't extreme, it's *precise*. The authors are advised to emulate this precision in their work.

19. **Line 500:** "who found" should be "which found".

20. **Line 503:** "Humans generally" should be "humans generally".

21. **Line 528:** "The model is clearly missing a key aspect of human walking, and it is useful to consider what that might be" It is not at all clear! Scientifically speaking, the model can be perfect but the experiment could be flawed.

22. **Fig. 6:** Here and throughout the paper statistical significance should be presented right along with the results.

23. **Line 608:** "... goal-directed updates a movement ..." should be "... goal-directed updates of a movement ...".

24. **Lines 618–649** This passage discusses the existence of a measure for the *degree* of stability. Regarding using the spectral radius $\rho(A)$ for this purpose, the authors write: "This is possible in principle, but it is questionable how useful such a definition would be in practice ...". The reason this is difficult in practice is that one doesn't have a validated theoretical model. If one did, then it would be a perfectly good—arguably the best—way to quantify the degree to which *small* disturbances are rejected (but not necessarily *large* disturbances). The argument of this paragraph is fundamentally flawed because the authors, in essence, claim that their parameter estimation results have conclusively invalidated the LIP model.

However, as mentioned in General Comment 8, they haven't actually done this. They've merely shown that their estimated parameters don't match the model's. They haven't thereby succeeded in showing that the model is categorically wrong. We can't take this one, rather preliminary, data analysis as being conclusive, one way or the other.

25. **Line 662–663:** "Humans live in a three-dimensional world and walk on mostly two-dimensional surfaces." *Mostly*? No, humans *always* walk on 2D surfaces. Moving in 3D "surfaces" is called flying.

26. **Lines 663–664:** "The approximation of the body as a single-link inverted pendulum allows to neglect the vertical dimension and analyze the two horizontal dimensions separately." This is incorrect. The vertical coordinate is only *approximately* constant for *linearized* pendulums.

27. **Lines 665–676:** This discussion is confused precisely in the way mentioned in Specific Comments 3 and 4. The entire system will only have *one* stability condition: the whole system, consisting of the differential equations acting in both directions, has a 4D phase space and is either entirely stable or unstable. It's true that in the LIP model the dynamics in the two directions are uncoupled and have the same governing equations (due to linearization), so each 2D subspace (in the 4D phase space) will have a similar stability triangle (Fig. 4). However, the parameters in the two directions *are not necessarily the same*. So, there is no real contrast with the work of Kuo: to translate the LIP model into the context of Kuo's 3D walker analysis, the lateral plane parameters would be expected to cause instability *before* the sagittal plane. Indeed, for Kuo's 3D walker, instability can occur in both frontal and sagittal directions, but the frontal direction is more critical for stable walking.

28. **Appendix 6.1:** The phrase "We derive the equation for $x(t)$ by time, getting ..." should be replaced with "We differentiate $x(t)$ with respect to time, getting ..." "Derive" refers to the very general process of doing analysis and algebra to obtain some result; it is not the same thing as "to differentiate" something. That said, I think this appendix can be eliminated. Eq. (2) is a very elementary differential equation that can be found in virtually any introductory textbook on the subject. It is sufficient to simply say that the displayed solution (the first equation in the section) can be shown to solve Eq. (2) "by direct substitution."

29. **Appendix 6.2:** This section involves the derivation of the maps that take (q_n, v_n) to (q_{n+1}, v_{n+1}). Some of the intermediate and very routine algebraic steps can be eliminated to get more quickly and cleanly to the last equation on p. 19 (the "progressive" map for q_{n+1}) and the last equation on p. 20 before line 712 (the "progressive" map for v_{n+1}). These two things should be given equation numbers because they're very important! Of course, the same is true for the "alternating" maps.

30. **Appendices 6.3–5:** Again, this all is presented in a very verbose and inelegant

manner that looks more complicated than it really is. In Appendix 6.3 one is simply finding the fixed point of the maps found in Appendix 6.2; linearizing about that fixed point is carried out in 6.4; and applying standard stability theory is done in 6.5. The entire presentation should be streamlined. Citations to authoritative texts on the topic can help in this process.

31. **Appendix 6.6:** The title says "Parameter estimates", but the necessary details of that process are not presented. How, *exactly*, was the estimation done? How was the quality of the fit assessed? As with the previous appendices, verbosity is not the goal, but enough information should be given so that others can duplicate your calculations. Frankly, this Appendix is more important than the large amount of elementary algebra in Appendices 6.2–5.

References

Patil, N. S., Dingwell, J. B., and Cusumano, J. P. (2020). Task-level regulation enhances global stability of the simplest dynamic walker. *Journal of The Royal Society Interface*, 17(168):20200278.

Patil, N. S., Dingwell, J. B., and Cusumano, J. P. (2022). Viability, task switching, and fall avoidance of the simplest dynamic walker. *Scientific Reports*, 12(1):8993.

Seydel, R. (2010). *Practical Bifurcation and Stability Analysis*, volume 5 of *Interdisciplinary Applied Mathematics*. Springer Science & Business Media, New York.

Zaytsev, P. and Ruina, A. (2003). Controllability and viability: a look at robustness of walking. *Experimental Brain Research*, 148:133–138.

Zaytsev, P., Wolfslag, W., and Ruina, A. (2018). The boundaries of walking stability: Viability and controllability of simple models. *IEEE Transactions on Robotics*, 34(2):336–352.
