# Supplementary material for: The condition for dynamic stability in humans walking with feedback control
Source: PLoS Comput Biol. 2024 Mar 18;20(3):e1011861. doi: 10.1371/journal.pcbi.1011861 (PMC10997112; doi:10.1371/journal.pcbi.1011861)
Supplement: Attachment — Submitted filename: responseToReviewers.pdf [file pcbi.1011861.s003.pdf]

We thank the reviewers for the extensive comments and helpful feedback. We have edited the manuscript based on the suggestions. This document contains detailed responses to each comment by the reviewers. The reviewer comments are numbered and bolded, our responses are in plain text and quotes from the updated manuscript are indented, with new text in blue and text that was removed in red and struck out. We submitted a new version with all changes marked, and a clean, updated version. Line numbers here refer to the version with changes marked, unless specified otherwise.

Reviewer #1:

1.1. This manuscript investigates an inverted pendulum model of walking with neural feedback control in the form of the stepping strategy. The concept is quite simple (and elegant) by combining two established methods – simple inverted pendulum models and foot placement control based on mid-stance. The description and development of the model were thorough and easy to follow. Although some theorems were unsurprising for those familiar with the models, the authors gave clear explanations for those that might be less familiar. The control gains and cadence finding is also very interesting and relevant to open questions in the field as well. The comparisons with human data as presented, however, left me unconvinced about the validity of the model in comparison to human behavior. Yet, the authors did an extensive job of outlining the utility and deficiencies of the model which was much appreciated.

Experimental data comparisons: Previous data was used to evaluate (very qualitatively) whether the model could predict human behavior. While I believe the human comparisons are probably necessary, they currently weaken the paper in my view. In Figure 5A, there is considerable scatter in the lower cadence data. While the shift in bd was later explained, the wider scatter in bp was not. In addition, comparing the model against human data at more than two cadences would be beneficial. A quantitative method of comparisons would also lend more strength to the arguments. I do not doubt the validity and usefulness of the model, but barring gathering more data, I would almost be more interested more broadly in the model's predictions, not just with respect to cadence.

We thank the reviewers for the generally positive comments. We share the reviewer's sentiments about the drawbacks expressed here. We agree that the fit with the experimental data is less than ideal. As discussed in the paper, we interpret this as an indicator that some substantial aspect of human walking is not captured yet by the model. Similarly, making both broader and deeper comparisons of model predictions to human data would be much better. In our view, this requires new studies with well-designed experiments to increase comparability between human walking behavior and the model as much as possible. Options for this would be to study walking and

multiple different cadences or speeds, or to remove participants who do not sufficiently follow the metronome to remove the scatter. Such comparisons, with new experimental studies, go beyond the scope of this paper. Our goal here was to show how such comparisons can be done and that they are useful in principle, by using data that we had available and a question that is reasonably interesting.

1.2. One notable feature missing from this model is collisional losses. As also noted in Lines 311-312, the model has no method of dissipating energy which can build up. A nice explanation was provided in the Discussion, but the argument for why the effect of collision is small is unconvincing. Although push-off can make up for this energy loss, collisional losses have a substantial effect on system dynamics. Impacts might also be another reason for the difference between the current model and the Bauby and Kuo model, rather than model complexity.

We partially agree with the reviewer here, but must admit that we simply do not know how big the influence of collisional losses are. As the reviewer will see, another reviewer of this manuscript (see 3.7) actually thinks that the effects of collisional losses are smaller than we expect them to be. Either way, the effects of such losses are hard to estimate, and most likely play a larger role when there is a substantial disbalance in push off-collisional loss (such as for instance as when facing AP perturbations. We also note that introducing collisions would substantially complicate the model (introduce several nonlinearities, which would make that we could no longer calculate return maps analytically), and hence, this is exactly what we refer to when we say that there is a difference in model complexity between Bauby and Kuo's model and ours (but, see also comment 3.S.27, and our reply). We have tried to better include these ideas in the text.

1.3. The sections on energy loss, effective leg length, and ankle roll were informative in outlining why model predictions might be wrong. It was also interesting that low bd could be explained by the lack of ankle roll. However, the effect of the other two missing items seemed to be treated more dismissively. On a similar note to the prior concern and without going outside the scope of the paper, could the authors give some indication whether a constant leg length is expected to have a large or small effect on model predictions? Otherwise, it currently reads that the ankle roll was already decided to have the largest effect based on the author' past work, which might be true, but minimizing the other two items still requires more support to be convincing to readers.

While we are certainly more knowledgeable about ankle roll as a mechanism for stability control, it was not our intent to treat energy loss and effective leg length dismissively.

We took up the reviewer's suggestion to briefly explore how a different effective leg length would affect the stability region and updated the section accordingly.

> The second reason why our model results show limited agreement with the human results is that we assumed the eigenfrequency of the body as an inverted pendulum, ω, to be constant. While this is a common assumption, the effective leg length, i.e. the distance between the body CoM and the contact point, changes substantially throughout the gait cycle. The common understanding is that the effective leg length is modulated to minimize vertical CoM motion [30], but modulation of the CoM height can also be used to affect the CoM dynamics, making it a candidate for a feedback control mechanism [34]. We are currently unaware of any experimental evidence that humans might actually use leg length modulation as a control mechanism during walking, in contrast to ankle roll (see below). In our comparison of model predictions with experimental data, we used the average effective leg length of the human participants, l = 0.77 m. The eigenfrequency parameter, $\omega = \sqrt{g l^{-1}}$, affects the stability of the system. Specifically, with increasing leg length the triangular stability region grows larger, and also shifts downward and to the left. This effect is relatively small, however, indicating that even if humans use active modulation of effective leg length as a control mechanism, the gain parameters for foot placement control would be largely unaffected by that. ~~so the location of the stability region changes with ω.~~ Here we note that not accounting for this relationship by using a single group estimate for ω might affect how the model prediction compares to the experimental data, but a detailed investigation of this effect would go beyond the scope of this manuscript.

1.4. Figure 5 – the caption notes that the human data in Figure 5BC is the same as from Figure 5A. Both are from a previous experiment where individuals walked at a set cadence dictated by a metronome. If so, why are there variations in the human cadences for Figure BC?

Unfortunately, our subjects were not always adhering to our instructions, and 'strayed' from the imposed metronome frequency. We have noted this in the figure caption now.

> The cadence for each participant is the average for that trial, which usually differs by some degree from the paces imposed by the metronome.

1.5. What was the approximate speed range at these two cadences? Were they comparable between human data and model? And among the studies discussed in "How is the model useful"?

We added the walking speeds of the human data to the text. The model does not have a walking speed in this case, since it is limited to the medial-lateral direction.

> … with resulting average walking speeds of 0.73 and 1.17 m s^{-1}.

1.6. Line 503 states that humans vary cadence "in direct proportion" to speed. However, there is also an established power law that links speed and step length (and cadence) by [1] (see also [2]), resulting in a more curvilinear relationship.

We used Inman's textbook as a reference. Reviewing the reference, we realized that we had made an error in this statement. The direct proportion observed in humans is between cadence and step length, rather than cadence and speed. Such a direct proportion between cadence and step length transforms to a power law relationship $s = \alpha \cdot v^{\beta}$ between speed, v, and step length, s, with an exponent of $\beta = 0.5$, which is within the range of 0.27–0.55 cited by Collins and Kuo (reference [2] provided by the reviewer). We apologize for this error and are glad the reviewer caught it. We adjusted the text accordingly (l. 545).

> Humans generally vary their cadence in direct proportion to their step length, and modulate both together to vary walking speed [30], so our model predicts an effect of walking speed that [13] did not observe.

Minor comments:
1.7 If cadence is to be the primary focus, it should be noted, perhaps in the last paragraph of Introduction.

We thank the reviewer for this suggestion, which we have incorporated into the introduction.

> Finally, we show how the theoretical model relates to actual walking by making predictions for how control parameters for stability in humans should change at different cadences and comparing them to experimental data.

1.8 Line 190 – how were the initial conditions chosen "exactly right"?

We did this by calculating the periodic orbit of the system, as shown in detail in Section 2.2. We added a pointer to this to the text.

> (we show below in Result 1 how to determine the periodic solution to find such an initial condition)

1.9 Line 572 – Typo ")although"

fixed

[1] D. W. Grieve, "Gait patterns and the speed of walking," Biomed. Eng., vol. 3, pp. 119–122, 1968.
[2] S. H. Collins and A. D. Kuo, "Two independent contributions to step variability during over-ground human walking," PLoS ONE, vol. 8, no. 8, p. e73597, 2013.

Reviewer #2

This manuscript addresses the very important topic of stability during human walking developing a simple model that incorporates both (bio)mechanics and neural control and then employing it to make predictions about the contribution of center of mass state feedback to dynamic balance at slow vs faster walking speeds. These predictions are tested using data from relevant human walking experiments in a laboratory setting.

First the authors clearly present mathematical descriptions of both the mechanical system (i.e., a two-legged, 2DOF (fore-aft + medio-lateral) passive inverted pendulum (Eqs. 1, 2)) and control system (i.e., a proportional-derivative feedback control law that maps center of mass (CoM) state at midstance to an updated contact point of the foot on the ground relative to the CoM (Eqs. 3,4) and show output of walking simulations driven by the model (Fig. 1).

Next, the authors mathematically define periodic orbits and their stability to changes in control gains (Figs.2, 3) culminating in the key result describing the range of feedback control parameters that yield stable walking for a fixed step time (Fig. 4).

Then, the authors test their model's prediction that slower walking (i.e., longer step time) should require higher feedback control gains against model-fits from human walking experiments at different walking speeds. The simple model does a reasonable job at predicting what humans do (Fig. 5).

Finally, the authors provide a comprehensive critique of their own model by addressing many, if not all, of the key limitations and how they might influence predictions about stability during real human walking (e.g., contribution of ankle roll to modulate CoP (Fig. 6). In doing so, the authors demonstrate how their model is 'wrong, and useful'- a welcome approach that is all too commonly lost in the literature in favor of presenting more complicated models with 'cleaner' results, often at the expense of deeper understanding of the system.

In short, the manuscript presents a 'first of its kind' simple neuro+mechanical model that, as the authors would say themselves, is 'wrong, but incredibly useful'. Their formulation acknowledges, and creatively merges previous mathematical descriptions of the mechanics and (neural) control of walking, with a keen focus on clearly and rigorously defining a set of formal conditions for dynamic stability. A strength is that the model is used to make testable predictions that are directly addressed with relevant human experiments. The interpretation of their results with respect to model limitations is especially strong. In this reviewer's opinion, this work is truly exciting and stands to have

great impact as a 'go to' foundation for how scientists, clinicians and engineers define, understand and work to preserve or augment balance in two-legged locomotion systems – not just in humans, but also robots, and humans wearing robots. The manuscript already offers a fine contribution as is, but below I offer a handful of comments that may help to further strengthen the clarity of the presentation and expand the scope of the discussion – two aspects that could increase the impact of the work.

We thank their reviewer for their praise and positive comments.

2.1. As stated above, the modeling approach to merge the mechanics and control of walking and application of formal analysis to define stability are both excellent and clearly described in text and using mathematical formulas. What is missing, however, is a clear schematic description of the elements of the model at the front-end of the main text. One potential version of this would show a 'cartoon' that defines the inverted pendulum geometry and states and a block diagram that represents the feedback control. Introducing the phase space representation in this schematic with an example of stable and unstable orbits would also help frame the reader's expectation early.

We agree with the reviewer that such a graphic would be a good idea, and have added it to the manuscript as Figure 1.

2.2. Along the lines of point 1. above, it would be helpful to have a clearer understanding of what the parameter b0 is? Is this akin to a baseline relative pose of the legs on the ground? i.e. a baseline 'base of support'? A pictorial representation of the system would help clarify this.

We hope that the addition of the model schematic (see 2.1) has helped here. We also added the following text to the methods to explain the role of this parameter.

> The additional parameter b_o is a constant offset to foot placement location. It can be used to modulate average step length or width, but, as we will show later, has no bearing on the stability of the system.

2.3. Linked to point 2. above, in some cases in the presentation of results it is unclear whether both A/P and M/L directions are being addressed (e.g., sensitivity to control parameters b0, bp, bd – Figs. 3, 4, 5). It is suggested to tighten and clarify throughout about whether/when AP vs. ML are being addressed. Perhaps using different subscripts or subplots?

We had clarifying text about this early in the methods section, but we realized that this might not be the ideal location. We replaced this with the following text at the end of the Control subsection, after the system has already been defined (l. 163–172).

> The only difference between the two versions of the system describing progressive walking in the anterior-posterior direction and alternating walking in the medial-lateral direction is the addition of the alternating sign of the offset. As we will see later, this has no effect on many system properties, including stability. In the interest of keeping notation simple and to avoid excessive subscripts in the analysis, we will generally only specify the direction of movement in cases where it is relevant. We also note that analyzing movement in the anterior-posterior and medial-lateral directions as separate, one-dimensional systems is possible due to the simplifying assumptions made here, specifically the linearization, lack of swing leg dynamics and constant step time. The dynamics of the actual system are, of course, three-dimensional.

We also added superscripts for the directions (ap, ml) in the equations for foot placement control (Equations 3, 4) and the reference states (Equations 7, 8). We hope that these two edits will sufficiently improve clarity.

2.4. Does it matter how the perturbation is applied? For example, is perturbing via a change in initial conditions (Fig. 1) the same as a perturbation from a fixed periodic orbit (Fig. 3)? Is a perturb in position the same as a perturb in velocity? Does the phase and direction of the perturbation matter? If so, how would these issues affect the stability analysis throughout?

This is an interesting, yet from our analyses unanswerable question. It should be kept in mind here that what we calculate is the local stability of a linearized system. Although we very much would like for this stability to have some relationship to the questions the reviewer poses here (i.e. what is the largest perturbation the model can handle, are there perturbations the model is more robust to than others, does the timing matter, etc.), these questions cannot be answered for the system analyzed here. We now explain this at the end of the subsection “How do the control parameters affect stability?” in l. 377–382.

> Note that while this result can tell us whether the walking system is asymptotically stable or not, this does not in any way relate to how large of a perturbation the system can handle. In fact, it has repeatedly been shown that there is a very limited (if any) relationship between the local stability of a system, in terms of eigenvalues around a fixed point or limit cycle, and robustness, in terms of the size of a perturbation that can be handled without falling, for walking systems [19–21].

2.5. In reading the manuscript, this reviewer noted many model simplifications that the authors would do well to address (e.g., massless limbs, no joints, no swing leg dynamics, no arms, no angular momentum!; -- no explicit force production of the legs on the ground, no double support period, no collision losses; -- a hyperfocus on midstance state as the discrete control input). But, by the end of the paper, the authors had handled most if not all of these – often pointing to model modifications that could add complexity if/when needed.

One aspect that could use a bit more discussion is how the feedback control law presented in this manuscript would work in the physiological system. What sensory modalities are involved, how are they integrated to give CoM position and velocity? What about integral control? (i.e. the ‘I’ in PID).

The reviewer is absolutely right that we largely “ignored” how the sensory information would be implemented in a physiological system. However, both of us have worked substantially on this topic, and thus, it is appropriate to shortly touch on these issues, which is what we now do. The following text has been added to the “limitations” section, l. 671–684:

> In fact, if humans actually estimate CoM state in some way, and how they do so, are still open research questions. While it is well known that visual [37,47], vestibular [48–51], and proprioceptive systems [52] all play a role in the control of foot placement (see also [18] for a review), it is unknown if, and how, these are integrated into an estimate of center of mass state. Nonetheless, in modeling work, center of mass state seems to predict balance responses better than joint level feedback, suggesting that sensory sources are integrated in some way [53].

2.6. The authors mention the need for ‘large sets of human data’ to ‘see the typical stability regions in humans’. There is a recent example of this, an open-source data set created by Jennifer Leestma at GaTech that may be useful to cite or even use in future collaborative work.

We wish to thank the reviewer for suggesting these datasets, and will keep these in mind for future work. Also, we appreciate the offer for future collaborative work, which we certainly will keep in mind when we continue working on these ideas (for now, however, we think that including more data would stray too far from the actual manuscript).

Reviewer 3

This paper is centered on the mathematical analysis of the local stability of periodic steady states of the well-known linearized inverted pendulum (LIP) model of human walking. A closed loop version of the LIP with position plus velocity feedback is the focus of the study, which also includes a comparison with experimental data. Such mathematical studies of the LIP and similar dynamic walking models are worthwhile and, in my opinion, should be done more frequently, as they have the potential to generate new insights into walking dynamics and issues of clinical interest, in particular the factors underlying fall risk and recovery. At the same time, mathematical analyses of walking models, when carefully compared to experimental data, can help us understand the limitations of individual models.

However, while this general aim of the paper is worthwhile and the core results are novel and have some merit, the manuscript suffers from an imprecise and, frankly, at times sloppy presentation of the material, inadequate engagement with the literature, and results that are not particularly coherent with the aims suggested by the introduction. Thus, I cannot recommend publication of the paper, at least not without a substantial rewrite and restructuring.

We are glad to hear that the reviewer shares our view that the aim of this paper is worthwhile. We have attempted to address the issues with the presentation and literature engagement they raise.

General Comments

3.1. The main results are presented in three "theorems". This is an inappropriate name for what are straightforward applications of standard local stability theory. In general, the word "theorem" is reserved for results of considerable importance and generality. The authors are, in fact, using a theorem to analyze stability of the fixed point of a map: namely, that all eigenvalues λ of the Jacobian of the map evaluated at the fixed point must have $|\lambda| \leq 1$ for stability, and $|\lambda| < 1$ for asymptotic stability. See, for example, Theorem 1.13 in Seydel (2010), section 1.4. Nor are these results "lemmas", which are results of lesser importance needed in the proof of a theorem. That said, I understand that the authors may want to label the results so that they can be referred to them later: I suggest simply calling them "Result 1", "Result 2",. . . , etc.

We thank the reviewer for this suggestion, which we have implemented.

3.2. Likewise, it is somewhat overblown to label as "proofs" the calculations needed to find the fixed points (periodic orbits) and assess their stability. People

who regularly do such work in dynamics and control would most likely simply say they were "calculating", "deriving", or "demonstrating" the results (or some similar language). Rather than labeling them as proofs in the appendices, they should just say "Derivation of Result 1", etc.

Like for comment 3.1, we agree with the reviewer, and have implemented as suggested.

3.3. Finally, while it is appropriate to display the main steps needed for these derivations in the appendices, showing so many steps of what are routine algebraic manipulations detracts from the paper. The inelegant mathematical presentation obscures the underlying simplicity of the analysis: those who already understand the basic theory find such a presentation tedious, whereas those who don't understand are not likely to find the many lines of algebra all that instructive.

We partially agree with the reviewer here. Although this is a modeling paper, the results might also be of interest for a non-technical audience, for whom these algebraic manipulations are less routine, as the reviewer also states, and we have included the details for these potential readers. However, those details are not necessary in the paper itself, and we have thus moved them to a Supplementary Materials document.

3.4. Please note that in the above comments I am not criticizing the results, only their presentation. (In fact, I confirmed the accuracy of the calculations myself.) The point here isn't pedantry, but to achieve clarity and to accurately represent the level of the authors' achievement.

We thank the reviewer for their comments, and for taking the time and effort of going through all the calculations themselves. As the reviewer will see, we have done our best to improve the manuscript along the lines suggested by the reviewer.

3.5. The strongest aspect of the paper is the stability analysis itself, culminating in the identification of the triangular stability region in the control parameter space (Fig. 4) and its dependency on cadence (Fig. 5). As summarized by the statement in lines 381–385:

> **These two examples indicate that the region of stable parameter sets shifts towards higher control gains for slower-paced walking. This means that when walking at a slower cadence, with longer step times, control gains need to be higher, or the system will not be stable. Similarly, when walking at faster stepping cadences, with shorter step times, control gains need to be lower.**

This is indeed an interesting observation! Unfortunately, the paper's pivot to testing against data seems to have distracted the authors from a more careful discussion in purely analytical terms. How precisely does their mathematical analysis compare/contrast with similar work (of Hurmuzlu, Hof, Ruina, Srinivasan, Kuo, Dingwell, to name a few just off the top of my head)? This is especially unfortunate since the second author is quite knowledgeable about such matters, having carried out a systematic review of gait stability analysis. How does this analytical work inform, clarify, correct, or move beyond previous such efforts?

We agree with the reviewer that the relationship to the existing literature was not well discussed in the manuscript. We added a section "Relationship to other modeling studies" to the discussion, where we remedy this. While not exhaustive, we attempted to cover the most relevant modeling studies from both robotics and human motor control, including several of the groups suggested by the reviewer.

3.6. The authors provide a partial response to the above question by pointing out that previous model-based efforts haven't tended to integrate biomechanical and neuromotor aspects of locomotion: this is largely true and an important observation. However, they neglect to note that exactly this perspective was taken in Patil et al. (2020, 2022), in which motor regulation and mechanical models were integrated to study not just local stability, but global stability and the even more general concept of viability introduced in Zaytsev et al. (2018); Zaytsev and Ruina (2003). In fact, whereas Patil et al. focus on a walker with swing leg dynamics, the work of Zaystev et al. includes a sophisticated stability analysis of the LIP that goes well beyond what's presented in this paper. Such work must be acknowledged and the relationship of this study to it and similar efforts should be discussed, particularly regarding what is novel about the authors' analysis.

Again, we agree with the reviewer on this point and apologize for neglecting this in the original manuscript. We have included these specific papers in the section "Relationship to other modeling studies" that we added to the discussion. We attempted to capture the main aspects of how these studies are similar/different from our work, and note that a more detailed analysis would be more suited for a separate review paper.

3.7. Returning to the comparison with experimental data: it's clear that the match isn't as good as one would hope, but I'd argue that it might be better than the authors realize. Their speculations on the sources of discrepancies focus on how:

(a) the model does not account for energy loss from collisions at heel strike;

(b) their analysis used a uniform effective leg length across all subjects; and

(c) the model does not incude the effect of ankle roll.

Of these, (b) is the most immediately relevant because it requires no change to the LIP model. Surely, including the correct leg length for each participant's data can and should be done. In contrast, as they point out, (a) would require a change in the model itself, at least during "heel strike". However, I don't believe the conservative nature of the model would have a large impact on this study: at steady state, the energy is in balance (as the authors point out), so near the steady state it will be close to being in balance and, thus, it shouldn't have a large impact on a local stability analysis. The issue of ankle roll, (c), would require a change in the model during the continuous-time swing phase. To their credit, they do check its importance by looking at data from trials with restricted ankle roll (Fig. 6), but they fail to show how this restricted data changes the plots of Fig. 5, which should be done. Furthermore, they don't say how the model would be changed to include this effect: would it be purely kinematic, or would some form of actuation, such as an ankle torque, be needed?

We share the reviewer's view that modeling (a) energy loss from collision would likely not change the results substantially. Modeling it explicitly would, however, substantially increase the complexity of the model, which is why we opted against it here, as discussed in the limitations section.

We have adopted the suggestion for (b) and normalized the experimental estimates by the leg length, specifically in Figure 6 (new numbering).

The absence of (c) ankle roll is indeed a simplification. The most straightforward way to add ankle roll to the model would be in the form of a continuous modulation of the CoP during the stance phase. We have opted against this for two reasons. (1) The structure of a control law for this "actuator" in humans is much less constrained by experimental data than for foot placement. While it is reasonably well established that humans use active torques to shift their CoP in the direction of a fall (see e.g. Reimann et al, Neural Control of Balance During Walking. Frontiers in Physiology. 2018), it is unclear how such a continuous control law is structured or coordinated with the gait cycle. (2) Adding ankle roll would make the model more complex, so that most of the results we derived analytically here would have to be replaced with numerical solutions. While we believe that ultimately (and, in fact, very soon) there is no way around a switch to numerical techniques, our goal here was to provide a solid foundation using analytical techniques. We have, in fact, already started working in the direction suggested by the reviewer, by exploring a continuous PD control law for CoP, and found that this implementation of "ankle roll" allows using derivative control gains for foot placement, b_d, that are below the stability region determined in Result 3. While this approach works well in principle, this work is not at a point where addition to the current manuscript would be feasible.

3.8. Beyond these sources of discrepancy, however, the authors miss several that are, in my opinion, likely to be as, or more, relevant:

(a) First and foremost, one should consider parameter estimation errors. The linear regressions used to estimate the Jacobians are not discussed in sufficient detail, particularly regarding their goodness of fit. It appears from Fig. 5A that the estimates of bp are reasonably good and are consistent with the hypothesis of cadence dependence (Fig. 5B). In contrast, the model consistently over-predicts the value of bd obtained from the data. When models disagree with data, the problem could be the model, it could be the data quality (it might be too imprecise or noisy, for example), it could be the data analysis, or it could be some combination of all three. One does not know a priori where the problem lies. The authors need to consider sources of error that lie entirely in the nature of the data and its processing.

The reviewer is correct that estimation errors may play a role. However, these analyses consistently yield high (often >0.9) R^2 values, which makes this problem relatively unlikely. This point has now been discussed in the limitations section (l. 678–684).

> The fact that high R^2 (up to 0.95) are consistently reported when estimating velocity and position gains from human data [13] suggest that these parameters can be estimated with good precision from the data. Here, we found R^2 values ranging from 0.75–0.98 for normal walking to a metronome at 110 beats per minute, and from 0.56–0.92 at for slow walking at 80 beats per minute, which suggests that the discrepancy in gains between the model and human data did not stem from a bad fit between the data and linear model.

(b) Second, the controller might be wrong. The fact that b_d is a poor fit might indicate that velocity feedback isn't being used (or is only weakly used). Indeed, if one sets b_d = 0, the controller is the same as Hof's "constant offset" model with position feedback, described in his 2008 paper cited by the authors. Perhaps it was a mistake to include velocity feedback in the first place?

Hof's "constant offset" control is a special case of the PD control law for foot placement described here, though in a different way than suggested by the reviewer. If one sets b_d = 0, the system is not stable, as shown by our Result 3 and the fact that the triangular stability region (Figure 5) does not overlap the horizontal axis (b_d = 0). Hof's constant offset control also includes velocity feedback, as he suggests to place the feet at a constant offset from the XCoM, which contains both position and velocity terms. We now make the relationship between our work and Hof's controller explicit in the discussion, in the new section "Relationship to other modeling studies" (l. 764–772).

Hof introduced the concept of the extrapolated center of mass [8] and suggested possible controllers to walking that maintain balance by modulating either the step time or the foot placement location [12]. For the latter, Hof proposed placing the foot at a constant offset from the extrapolated center of mass. This controller can be expressed as a special case of the control law analyzed here (Equations 3, 4), with control gains bp = c + s and bd = ω−1(c + s), where some transformations are required due to Hof using the state at foot placement, rather than at midstance, as input. These parameters are well within the stability region (see Figure 5), with spectral norm ρ = 0.21 for the example used here.

(c) Third, the model uses a constant value of the the parameter p (described as "the contact point between the leg and the ground", line 121). Could it be that this needs to be taken as time dependent during each step?

Freeing the contact point, p, up for continuous modulation would be equivalent to modeling modulation of the CoP, e.g. using the ankle roll mechanism, as described above in our response to point 3.7 (c).

3.9. Finally, the authors spend a significant portion of the introduction talking about Hof's XCoM and MoS concepts, stating: "We postulate that the main reason that the MoS does not work well as a stability measure in walking is that it neglects the control aspect." But, while the Discussion does again mention MoS, the authors never return to examine their postulate. Does their work in some way resolve the "paradoxes" they refer to? If that isn't an aim of the paper, why focus on it so much in the introduction?

This is a fair point, and we thank the reviewer for pointing it out. We focused on the MoS in the introduction because this is currently a topic of debate, and we believe that the underlying issue with it is, indeed, that it is purely biomechanical. We did not, on the other hand, provide any alternative in the current manuscript, although we hope that our work might be useful as the basis for one. We added the following text to the discussion to make this explicit (l. 529–535).

We stress that in the model analysis presented here only answered the first question. We did not, in any way, provide a measure for a degree of stability. Specifically, our work does not offer any measure that is applicable to experimental data in the way the MoS is. While the MoS has several drawbacks, as discussed in the introduction, it is currently the most useful way we have to quantify stability of actual walking humans.

We also made several smaller changes to the parts of the introduction discussing the MoS to clarify our position and the contribution of this paper (l. 50-61) .

Specific Comments

3.S.1. Lines 48–52: Other than that the authors are making a distinction between standing and walking, I don't understand this passage, especially the part about how "the qualifier is not useful". What qualifier?

The qualifier is "without taking a step". We added text to make this explicit (l. 50). The main point is that a negative MoS means that the body cannot be kept upright without taking a step, which is a meaningful distinction in standing, but less so in walking.

3.S.2. Fig. 1 and lines 200–206: The authors say the red and yellow solutions are the same as the blue solution. They certainly don't look the same!

We are unsure what the reviewer means here, as we do not state that the solutions are the same. Actually, line 200 [original line numbers, l. 219 now] reads: "This shows that although the red and orange solutions are different, they relax to the same periodic orbit after some time, in relative terms." To clarify, the *system* is the same here. The three *solutions* have different initial conditions and thus lead to different trajectories. These trajectories relax to the same limit cycle.

3.S.3. Fig. 2, Theorem 1, and supporting discussion throughout: Like Hof (and others), the authors treat the frontal and sagittal directions as uncoupled and independent LIPs. I also understand why the authors have chosen to use x and v for position and velocity in both directions, namely, to keep the notation simple. However, the downside of this is the possibility of conceptual confusion. Specifically, one must realize that Eqs. (3) and (4) are not part of two separate systems. The lateral dynamics can't have different periodic orbits than the sagittal dynamics: they're both happening together in the same 4D phase space with states that might be written as (xA, vA, xP , vP), where subscripts A and P refer to the "alternating" and "progressive" components of the system, respectively. So, in particular, Theorem 1 is misleading if not interpreted carefully: the overall periodic gait must have period 2Tstep, even though the forward ("progressive") direction repeats every Tstep time units.

We clarified the direction reference by adding superscripts (ap, ml) in the equations for foot placement control (Equations 3, 4) and the reference states (Equations 7, 8). We hope that these two edits will sufficiently improve clarity. We also added the following text to clarify how the model relates to the physical system (l. 163–172).

> The only difference between the two versions of the system describing progressive walking in the anterior-posterior direction and alternating walking in the medial-lateral direction is the addition of the alternating sign of the offset. As we will see later, this has no effect on many system properties, including stability.

In the interest of keeping notation simple and to avoid excessive subscripts in the analysis, we will generally only specify the direction of movement in cases where it is relevant. We also note that analyzing movement in the anterior-posterior and medial-lateral directions as separate, one-dimensional systems is possible due to the simplifying assumptions made here, specifically the linearization, lack of swing leg dynamics and constant step time. The dynamics of the actual system are, of course, three-dimensional.

3.S.4. Continuing with the above comment, the control gains bp and bd were selected to be the same for both directions in simulations (only the offset b0 is different). There's no problem with this if one merely wants to study qualitatively reasonable- looking CoM trajectories, but there's no a priori reason to think they would be the same in a human. In this analysis, motion in the two directions (front to back or "progressive" vs. side to side or "alternating") will both be stable or unstable at the same time. But why should we assume that's the case? Moreover, what impact would such a difference have in Figs. 4 and 5?

We agree with the reviewer that bp and bd need not be equal in both directions. We selected the same parameter values for both systems in the example simulations in Figure 1 (old figure numbers) for the sake of simplicity. When analyzing the two directions as sub-spaces of a single system, then the parameter pairs for both directions must fall within the stable region in parameter space for the overall motion to be stable. In Figures 4 and 5 we do not "select" any single parameter value for b_p and b_d, but rather analyze the parameter space as a whole. Specifically, we do not assume that the values would be identical for the two directions of a 2d system. We ask the reviewer to explain further in case we misunderstood.

3.S.5. Line 266: "Note that this limit line is only a choice of the illustration." I don't understand what this means. What choice is being made?

We refer to the choice in the figure design of which flow lines to show on which area. We added the gray demarcation line to show where the flow lines change from one system to the next due to the foot placement changing p_0 to p_1. We added text in lines 285–291 to clarify this.

This change in dynamics is indicated by the vertical gray lines. Note that this limit line, where the illustration changes from showing the flow lines of the system for p_0 to the system for p_1, is only a choice of the illustration. The change of dynamics~~, illustrated by the flow lines,~~ is a result of the change in foot placement, which happens at a specific point in time, rather than when the CoM position, x, reaches the vertical line. At this point in time, the whole system dynamics changes, including the parts beyond the area shown in Figure 3.

3.S.6. Line 277: The line stops in the middle of a sentence.

fixed.

3.S.7. Section 2.3 Stability: The authors need to make it clear that they're focusing strictly on local or linear stability, which deals with how the system responds to sufficiently small perturbations of its state. As pointed out in General Comment 6, there are other, more general notions of stability (global stability, viability, possibly others) being examined by researchers studying bipedal walking. The authors don't have to do anything with these other definitions, but they do need to state that they're looking only at local stability. There's nothing wrong with doing that, but clarity is required. Likewise, they should include this as a significant limitation in the discussion starting on line 591.

We have added the following text to the beginning of Section 2.3 (l. 302–304). We also cover this in the new section "Relationship to other models" added to the discussion (see comment 3.5).

> We analyze the local stability of the walking system, i.e. how it responds to small perturbations. Note that this is conceptually different from related concepts such as global stability or viability [19].

3.S.8. Line 290–291: "As implied by Theorem 1, each walking system has exactly one periodic orbit." The way it is constructed, this is not what Theorem 1 does. It finds a periodic orbit. It does not show that that is the only periodic orbit.

We fixed this throughout the manuscript.

3.S.9. Theorem 2: This is not quite correct. The system is stable when the eigenvalues of A are ≤ 1; it's asymptotically stable when they're strictly < 1. Thus, the transition case occurs when they equal 1 exactly.

This is a terminology issue. Right before Theorem 2 we had defined "stable" for a walking system to mean that the system converges to the reference state after small deviations, which is synonymous with "asymptotic stability" in the dynamical systems sense. Our goal was to simplify terminology, but we realize now that our choice might have had the opposite effect for some readers. We edited the text to add "asymptotocally" here and in some other instances to clarify.

3.S.10. Fig. 4 and related discussion: First, the word "eigenvalue" should not be capitalized if it's not at the beginning of a sentence. Second, at first glance, from Fig. 4 one gets the impression that the authors are using ρ to be an eigenvalue of A. However, they don't at this point define the symbol, so we have to guess at its meaning. It's not until 13 pages later that they say that ρ(A) is the spectral radius,

which is the largest absolute value (or magnitude) of the eigenvalues. Eigenvalues and spectral radius are related, but not the same thing. One might suspect that the authors have confused the two. A better (and conventional) way to to present this is to say that the eigenvalues of A are λ1 and λ2, and ρ = max(|λ1|,|λ2|) is the spectral radius. This should be stated clearly in or before Theorem 2 and in Fig. 4's caption.

(1) We changed eigen to lower case in all instances of eigenvalue and eigenfrequency. We apologize for the first author's Germanness breaking through in the urge to capitalize words with this unmistakable German prefix.

(2) We added explanation of the symbol ρ to the figure caption

We chose not to introduce symbols for the eigenvalues at this point, because they are not used in any equations yet, and rather refer to them in plain language. We assure the reviewer that we are aware that eigenvalue and spectral norm are two different things. We assume that the reviewers impression was created by the term "largest absolute eigenvalue ρ(A)", which the reviewer might have read as "Eigenvalues are named ρ, and we take the largest absolute one", while we intended it as "we take the largest absolute Eigenvalue and call it ρ". We clarified this by adding the following explanation in l. 387.

> Color represents the largest absolute eigenvalue of A, which is referred to as the spectral norm, ρ(A).

3.S.11. Line 356: Same problem as above. "Color represents the largest absolute Eigenvalue ρ(A)" should be rewritten as "Color represents the value of the spectral radius ρ(A), which is the largest absolute value of the eigenvalues of A."

See 3.S.10 above.

3.S.12. Section 3: It makes more sense to say "Predictions and Results", since predictions come before the results.

We have changed this heading to "predictions and comparison to human data", as we use the term "results" now for what where previously our theorems.

3.S.13. Fig. 5: The estimated parameter values results are presented, and for details the reader is referred to Appendix 6.6. However, that appendix just gives the calculus for the Jacobian. It doesn't say how the data was processed to perform the regression estimates, nor does it talk about how their quality was assessed (i.e., goodness of fit estimates).

We now describe this in more detail, but for detailed descriptions do refer to our earlier published works. The text now reads (Supplementary Materials, l. 65–69)

> We calculated these slopes relating the variations in mediolateral center of mass position and velocity during midstance to variations in mediolateral foot placement (at heelstrike), as described in many of our previously published papers cite [39, 40, 42]. The goodness of fit was assessed via the R^2, and typically exceeded 0.7.

3.S.14. Continuing from the above comment, it's important to explain how the data in the two movement directions were processed. Where both sagittal plane and frontal plan components merged to estimate one pair (bp, bd)? Or were the gains estimated independently in both directions? This is yet another possible source of observed discrepancies between model and data.

We only analyzed the medial-lateral direction here. We now clarify this in line 442.

> To see how model predictions agree with actual human walking, we calculated the slopes, bp and bd, for the medial-lateral direction from an existing data set from a previous experiment.

3.S.15. Lines 406–408: here and throughout, the estimated values of bp and bd are referred to as "slopes". It would be more clear to simply call them parameter estimates. A standard notation in such cases is to say bp is the estimated value of bp, etc. Introducing new regression parameters βp and βd (what the authors call the "slopes") and then saying these are, in fact, equal to the estimated values of bp and bd is completely unecessary.

We agree with the reviewer and replaced β with $\widehat b$ throughout the manuscript.

3.S.16. Lines 429–430 and 525: "Overall, this shows that the model predicts some aspects of human stability control with foot placement . . . " What aspects? This is never made clear!

We are sorry for being vague here. The text has been rephrased:

> Overall, this shows that the model predicts the position gains observed in humans reasonably well, especially for higher cadence walking. Moreover, the model showed the same trend for increasing cadence as found in humans. However, velocity gains were less well predicted by the model.

3.S.17. Line 466–474: This passage is confusingly written and, frankly, wrong. Obviously people take steps when walking. The MoS does not specify "how close

the biomechanical state of the system is to the threshold of having to take a step" because people are continuously "having to take steps" when walking! The MoS specifies when people would have to take specific actions to avoid falling while taking steps during steady gait (such as crossover steps, or shifting their CoM with their torso and arms, etc.).

We are confused by the reviewers confusion and will do our best to clarify based on our reading of the comment. We follow Hof's definition of the MoS as the "minimum distance from XcoM to the boundaries of the BoS" (Hof et al., 2008, [5] in the references). We assume the convention that the MoS has a negative sign when the XCoM is outside the BoS. Then, a positive MoS means that the XCoM is within the base of support, so the CoP can be moved onto the XCoM, using mechanism like ankle roll or counter-rotation of the upper body. This "captures" the inverted pendulum by letting the point mass come to rest directly above the CoP. A negative MoS, on the other hand, means that the body mass cannot be captured without changing the base of support, i.e. taking a step. A MoS of zero is the threshold of having to take a step, because the biomechanical state of the body changes between not having to take a step at positive MoS to having to take a step at negative MoS values. This makes the MoS a useful number for standing, because *taking a step* is an important event that usually does not happen during standing. On the other hand, *taking a step* is a commonplace event during walking, so interpreting the MoS is less straightforward.

We hope that the reviewer can clear up the source of their confusion based on this explanation, so we can clarify in the main text if they deem appropriate.

3.S.18. Lines 475–478: This is the first place the authors mention asymptotic stability. It should be mentioned much earlier, certainly before/while presenting the methods. And what on Earth is "mathematical extremism"? I'd say the theory of stability isn't extreme, it's precise. The authors are advised to emulate this precision in their work.

We respectfully disagree with the reviewer here. We actually mention asymptotic stability when talking about the stability of the model right after introducing equation 10, where it reads: "Note that this definition of stability is the same as the asymptotic stability of the periodic orbit."

3.S.19. Line 500: "who found" should be "which found".

fixed

3.S.20. Line 503: "Humans generally" should be "humans generally".

A full stop should have been added to the sentence before that, which we did now.

3.S.21. Line 528: “The model is clearly missing a key aspect of human walking, and it is useful to consider what that might be” It is not at all clear! Scientifically speaking, the model can be perfect but the experiment could be flawed.

We agree with the reviewer and edited the text accordingly (l. 571–574):

> Our modelling results and the experimental data clearly do not fully match, and it may be interesting to consider why this is so. This mismatch could either be due to the experimental design introducing unintended effects, e.g. from walking on a treadmill, or from the model missing some key aspects of human walking.

3.S.22. Fig. 6: Here and throughout the paper statistical significance should be presented right along with the results.

We listed the p-value for the statistical test for this result along with it in the text (l. 639, l. 641).

3.S.23. Line 608: “. . . goal-directed updates a movement . . . ” should be “. . . goal-directed updates of a movement . . . ”.

Fixed

3.S.24. Lines 618–649 This passage discusses the existence of a measure for the degree of stability. Regarding using the spectral radius ρ(A) for this purpose, the authors write: “This is possible in principle, but it is questionable how useful such a definition would be in practice . . . ”. The reason this is difficult in practice is that one doesn’t have a validated theoretical model. If one did, then it would be a perfectly good—arguably the best—way to quantify the degree to which small disturbances are rejected (but not necessarily large disturbances). The argument of this paragraph is fundamentally flawed because the authors, in essence, claim that their parameter estimation results have conclusively invalidated the LIP model. However, as mentioned in General Comment 8, they haven’t actually done this. They’ve merely shown that their estimated parameters don’t match the model’s. They haven’t thereby succeeded in showing that the model is categorically wrong. We can’t take this one, rather preliminary, data analysis as being conclusive, one way or the other.

We did not intend to claim that our results conclusively invalidated something as useful as the LIP model. Our goal with this paragraph was to clearly lay out the reasons why, in the current state, our model and the associated parameters are not good candidates for stability measures that would be useful in the analysis of experimental data. We clarified this by adding the following sentence to the end of this paragraph (l. 717–719).

For these reasons, while a stability measure based on this model, or a similar one, could be very useful, such a model would have to be fully validated and explain the human data more closely than the version presented here as a starting point.

3.S.25. Line 662–663: "Humans live in a three-dimensional world and walk on mostly two-dimensional surfaces." Mostly? No, humans always walk on 2D surfaces. Moving in 3D "surfaces" is called flying.

We changed to "Humans live in a three-dimensional world and walk on two-dimensional surfaces". We had originally, in an effort to be precise, added the "mostly" to allow for special cases where the third dimension becomes relevant, such as walking on stairs, curved planes or on soft, yielding sand.

3.S.26. Lines 663–664: "The approximation of the body as a single-link inverted pendulum allows to neglect the vertical dimension and analyze the two horizontal dimensions separately." This is incorrect. The vertical coordinate is only approximately constant for linearized pendulums.

Our model is based, among others, on Kajita's work (Kajita et al., 2003, [16] in the reference list), using a horizontal constraint plane for the point mass, which yields the equation of motion we use for the LIP in this manuscript. In a horizontal plane, the vertical dimension is, by definition, constant. We do not understand which aspect of that is only approximal and respectfully ask the reviewer to elaborate.

3.S.27. Lines 665–676: This discussion is confused precisely in the way mentioned in Specific Comments 3 and 4. The entire system will only have one stability condition: the whole system, consisting of the differential equations acting in both directions, has a 4D phase space and is either entirely stable or unstable. It's true that in the LIP model the dynamics in the two directions are uncoupled and have the same governing equations (due to linearization), so each 2D subspace (in the 4D phase space) will have a similar stability triangle (Fig. 4). However, the parameters in the two directions are not necessarily the same. So, there is no real contrast with the work of Kuo: to translate the LIP model into the context of Kuo's 3D walker analysis, the lateral plane parameters would be expected to cause instability before the sagittal plane. Indeed, for Kuo's 3D walker, instability can occur in both frontal and sagittal directions, but the frontal direction is more critical for stable walking.

The contrast between our work and Kuo's lies in the fact that our model predicts that stability is the same in the frontal and the sagittal plane, while Kuo, and others, find that it is not. We would call this contrast "real", in that our results predict the opposite from what Kuo's models predict and the experimental data show. We assume that by "not

real", the reviewer intended to express that the mismatch is due to specific assumptions and (over-)simplifications made in our model, and we readily agree with that. We edited the text as follows to clarify (l. 748).

> Our model fails to predict this difference between frontal and sagittal plane stability. We postulate that the reasons for this failure ~~difference between results from the model presented here and these results from other models and the literature~~ are due to the various factors we neglected in the model.

The reviewer states that "for Kuo's 3D walker, instability can occur in both frontal and sagittal directions, but the frontal direction is more critical for stable walking.". This underlines our point, because Kuo found that in a 3d passive walker, the long-period gait has one unstable mode, corresponding to a roll motion in the frontal plane. This is in contrast to the planar walker, which is passively stable. So in this particular model, which we referenced, instability can occur only in the frontal plane by our understanding. We hope that the reviewer can clarify if there is a misunderstanding or they referred to a different model.

3.S.28. Appendix 6.1: The phrase "We derive the equation for x(t) by time, getting . . . " should be replaced with "We differentiate x(t) with respect to time, getting . . . " "Derive" refers to the very general process of doing analysis and algebra to obtain some result; it is not the same thing as "to differentiate" something. That said, I think this appendix can be eliminated. Eq. (2) is a very elementary differential equation that can be found in virtually any introductory textbook on the subject. It is sufficient to simply say that the displayed solution (the first equation in the section) can be shown to solve Eq. (2) "by direct substitution."

We corrected "derive" → "differentiate".

We agree with the reviewer that this equation is elementary for a certain audience. We added it for a different audience, with less of a technical background, who are not used to differential equations and conceiving of a function as a whole as a solution to an equation. We believe that changing all appendices to the supplementary materials hides this content sufficiently from the part of the audience it isn't intended for.

3.S.29. Appendix 6.2: This section involves the derivation of the maps that take (qn, vn) to (qn+1 , vn+1). Some of the intermediate and very routine algebraic steps can be eliminated to get more quickly and cleanly to the last equation on p. 19 (the "progressive" map for qn+1) and the last equation on p. 20 before line 712 (the "progressive" map for vn+1). These two things should be given equation numbers because they're very important! Of course, the same is true for the "alternating" maps.

We added equation numbers at the suggested locations. We kept the intermediate steps for the same reason given above in 3.S.28.

3.S.30. Appendices 6.3–5: Again, this all is presented in a very verbose and inelegant manner that looks more complicated than it really is. In Appendix 6.3 one is simply finding the fixed point of the maps found in Appendix 6.2; linearizing about that fixed point is carried out in 6.4; and applying standard stability theory is done in 6.5. The entire presentation should be streamlined. Citations to authoritative texts on the topic can help in this process.

See our response to 3.S.28 above. Complexity is relative, and while to a technically trained reader these things are straightforward, the intended audience includes different fields. Again, we hope that moving these details to the supplementary materials is an acceptable solution.

3.S.31. Appendix 6.6: The title says “Parameter estimates”, but the necessary details of that process are not presented. How, exactly, was the estimation done? How was the quality of the fit assessed? As with the previous appendices, verbosity is not the goal, but enough information should be given so that others can duplicate your calculations. Frankly, this Appendix is more important than the large amount of elementary algebra in Appendices 6.2–5.

We hope that our response to comment 3.S.16 has resolved this issue.
