# Supplementary material for: The condition for dynamic stability in humans walking with feedback control
Source: PLoS Comput Biol. 2024 Mar 18;20(3):e1011861. doi: 10.1371/journal.pcbi.1011861 (PMC10997112; doi:10.1371/journal.pcbi.1011861)
Supplement: Attachment — Submitted filename: PCOMPBIOL-D-23-00629_R1_review.pdf [file pcbi.1011861.s004.pdf]

Review: *PLOS Computational Biology*, PCOMPBIOL-D-23-00629_R1

Title: The condition for dynamic stability in humans ...

Authors: H. Reimann and S. Bruijn

The authors have responded to my and the other reviewers' criticisms and made a substantial number of changes to their manuscript to address them. They have modified the paper to clarify the nature and scope of their study and how their conclusions now more clearly follow from it. In particular, they have now clarified the type of stability analysis they do and its limitations, and do a better job relating it to similar and related studies in the literature.

I still believe the derivations are excessive given their elementary nature. However, the authors have moved them to supplemental material, which is an improvement, and they are are no longer given exaggerated significance by calling them "theorems". So I'm comfortable with the authors doing what they think is best in this instance: given that the derivations are, at least, technically correct, this is a stylistic matter.

There are a few problems that remain, however: a few of these are responses to the authors' questions to me; others mainly involve continued logical inconsistencies and conceptual confusion about the nature of their system model. All of these are fairly easy to address, with only relatively small edits required in each case.

My comments are itemized using the labels from the authors' responses.

3.8(a) The paper now includes R^2 estimates, but a few issues remain:

1. Citing values from other studies is not particularly relevant. We can't compare experimentalists and data in this way: one group's experimental results might simply be better than another's. At most, such citations indicate that it's *possible* to get good experimental results. It has no bearing on whether the results of this particular paper are accurate.
2. What does the range mean? We don't have a sense of how many of the estimates were near which end of the range. Why not show a box plot of the R^2 values? At least give us the mean/median and some mention of variance or confidence intervals.
3. Most importantly, the R^2 is for the simultaneous estimate of both parameters. This misses the main point of my original comment, which was about each parameter *individually*. That is, my main question was about whether or not both the proportional and derivative controllers are needed to capture your data. So, the question is: how much variance do b_d and b_p *each on their own* capture? The fact remains that b_d fits the data very well at both walking speeds, whereas b_p does not.

3.S.3 In responding to my comment on the four-dimensional nature of the system's phase space (or state space), the authors have added a paragraph starting

with "[t]he only difference between the two versions of the system describing progressive walking in the anterior-posterior direction and alternating walking in the medial-lateral direction is the addition of the alternating sign of the offset;" and concluding with "[t]he dynamics of the actual system are, of course, three-dimensional."

Unfortunately, this paragraph suggests that the authors may still not have clarity about the actual nature of the system they're looking at. First, there is only *one* system with *one* center of mass, not "two versions" of the system. Each alternating step and each progressive step is taken at the same time. Second, when they say that the system is "three dimensional", they seem to be confusing the dimension of *physical space* the walker moves in, which is what they mean by three, with the dimension of the walker's *state space*, which is four. The latter is what my comment was referring to. The authors have glossed over this aspect of my comment. On top of that, in saying that the walker moves in three physical space dimensions, the authors contradict their other statements elsewhere in this document (**3.S.26**) that it moves in a plane (which is true–to leading order, the CoM moves in the horizontal plane).

3.S.3 I understand that the triangle plots don't require the parameters to be the same. That was precisely my point: the different directions do not have to lose stability together. It's possible, for example, for the progressive direction to be stable and the alternating direction to be unstable. Yet, the manuscript says, in referring to Kuo's 3D walker (lines 731–732 of the revised manuscript): "Our model fails to predict this difference between frontal and sagittal plane stability." This is false. Just as with Kuo's model, your model can lose stability laterally and not in the sagittal direction if parameters are chosen to permit it. See also my responses to **3.S.4** and **3.S.27**.

3.S.4 This is a continuation of the issue discussed in my response to **3.S.3**. The problem is the authors keep saying their stability results are different from those found with Kuo's 3D walker. But they also say: "Specifically, we do not assume that the values would be identical for the two directions of a 2d system". Good! Perhaps what they're trying to say is that the stability *analysis* is identical in both directions, which is true—but the actual *stability* doesn't have to be the same because the parameters don't have to be the same. Thus, when you say that this is different than Kuo's model you are being logically inconsistent and, therefore, incorrect. Your model allows each direction to be stable or unstable independently of the other, depending on the parameter values chosen, so you can make it behave the same as Kuo's model. In other words, the model doesn't just have two parameters, $\{b_p, b_d\}$, it has *four*, which might be written $\{b_p^A, b_d^A, b_p^P, b_d^P\}$, where the superscripts A and P indicate the alternating and progressive directions, respectively.

3.S.17 The authors are "confused by my confusion", so let me be more direct: it is

absurd to say that MoS defines "when people will have to take a step" when applying it to gait, during which people *always* have to take steps to keep walking. The authors justify this description by applying the MoS concept to the task of standing, which is a poor paradigm to use for walking. Yet, the solution to the problem is right there, when they quote Hof: "the MoS is the minimum distance from the XCoM to the boundaries of the BoS." That's it! That's what they should say the MoS is because that's its definition. When walking, if the XCoM exceeds the BoS, the mass center will overshoot the base of support (defined by the current stance foot) before the next step occurs.

3.S.18 The authors remain attached to the notion that stability theory is "mathematical extremism." On lines 497-504 of revised manuscript, they say [underlining mine]:

> In dynamical systems theory, asymptotic stability is a well-defined property of a fixed point or periodic orbit of a system [25]. In typical mathematical extremism, this defines a "substantial change" as any change at all following an infinitesimally small perturbation after an infinitely long time. While extreme, this definition and associated methods from dynamical systems theory have also been widely applied to the analysis of walking movements, mostly in the form of measures that quantify how fast a system relaxes back towards the stable state after a perturbation, e.g. Lyapunov exponents [26], Floquet multipliers [27].

I'd say that that the mathematical description of stability presented by the authors may sound "extreme", but that's only because they have done such a poor job of it. Perhaps they are deploying a straw man argument, in an attempt to criticize work they feel is overly mathematical. Or, they might simply be attempting to be humorously descriptive. Maybe it is just unclear writing. I certainly can't divine their motivations. But, intentionally or not, they obscure the relatively simple meaning of stability. If the authors really want to reach non-experts (which they claim they want to do, and is why they want to include the elementary math now moved to the supplemental material), it is a disservice to that community to confuse them with an inadequate description of such an important concept.

Stability can be understood without boggling people's minds. For example, it took me thirty seconds to find this simple, nonmathematical description on Wikipedia:

> Stability theory addresses the following questions: Will a nearby orbit indefinitely stay close to a given orbit? Will it converge to the given orbit? In the former case, the orbit is called stable; in the

> latter case, it is called asymptotically stable and the given orbit is said to be attracting.

There's this more authoritative plain-English description on Scholarpedia:

> The stability of an orbit of a dynamical system characterizes whether nearby (i.e., perturbed) orbits will remain in a neighborhood of that orbit or be repelled away from it. Asymptotic stability additionally characterizes attraction of nearby orbits to this orbit in the long-time limit.

Notice neither of these refer to "infinitely long times" or "infinitesimally small perturbations". They are technically correct but accessible to non-experts.

This need not be difficult. Frankly, another option for the authors would be to simply remove most of the underlined portions of the above-quoted text from the manuscript. The remaining paragraph would still be perfectly serviceable.

3.S.26 The authors state: "In a horizontal plane, the vertical dimension is, by definition, constant. We do not understand which aspect of that is only approximal and respectfully ask the reviewer to elaborate." The authors join all other researchers in referring to their model as an inverted pendulum. However, the pendulum is a nonlinear mechanical system. The mass at the tip of an inverted pendulum can obviously change its height, as is obvious if one looks at a picture. It is only for small angular deflections (or, equivalently, small displacements) that the tip mass can be said to *approximately* not change its height and to stay *approximately* in a horizontal plane. That is, the inverted pendulum model the authors (and Hof, and others) use is a *linearized* inverted pendulum, obtained by Taylor series expansion about the vertical position. The vertical position is only constant to leading order in the displacements.

3.S.27 I have already discussed this multiple times. The authors state: "The contrast between our work and Kuo's lies in the fact that our model predicts that stability is the same in the frontal and the sagittal plane, while Kuo, and others, find that it is not." As stated in my responses to **3.S.3** and **3.S.4**, this is false. Your model is perfectly capable of having different sagittal and frontal plane stability. All you have to do is set the parameters differently in the two directions so that this occurs.
