# Supplementary material for: The condition for dynamic stability in humans walking with feedback control
Source: PLoS Comput Biol. 2024 Mar 18;20(3):e1011861. doi: 10.1371/journal.pcbi.1011861 (PMC10997112; doi:10.1371/journal.pcbi.1011861)
Supplement: Attachment — Submitted filename: responseToReviewers.pdf [file pcbi.1011861.s005.pdf]

We again thank the reviewers for the comments and helpful feedback and have edited the manuscript based on the suggestions. This document contains detailed responses to each comment by the reviewers. The reviewer comments are numbered and bolded, our responses are in plain text and quotes from the updated manuscript are indented, with new text in blue and text that was removed in red and struck out. We submitted a new version with all changes marked, and a clean, updated version. Line numbers here refer to the version with changes marked, unless specified otherwise.

Reviewer #1:

The authors have addressed my concerns in their thorough responses. I have a few minor suggestions.

We addressed all remaining concerns and thank the reviewer for their effort.

Figure 1 is a very helpful overview, but a more detailed caption might also help the reader to define some of the variables or know broadly what the components mean without searching in the text.

We agree with the reviewer and added a more detailed caption.

I do not think my comment about data accessibility was relayed to the authors, so I have reiterated it here. The human data is currently inaccessible, e.g., cannot access "foot_placement_ml_by_com_50_CAD.csv" when running the Matlab code.

We added the data files to the github repository (github.com/hendrikreimann/WalkingModelPaper2023) and apologize for this oversight.

Reviewer #2:

Great job addressing feedback in the revised submission. Looking forward to citing this when it is published.

Nice work.

We appreciate the kind words and thank the reviewer for their effort.

Reviewer #3:

The authors have responded to my and the other reviewers' criticisms and made a substantial number of changes to their manuscript to address them. They have

modified the paper to clarify the nature and scope of their study and how their conclusions now more clearly follow from it. In particular, they have now clarified the type of stability analysis they do and its limitations, and do a better job relating it to similar and related studies in the literature.

I still believe the derivations are excessive given their elementary nature. However, the authors have moved them to supplemental material, which is an improvement, and they are are no longer given exaggerated significance by calling them “theorems”. So I’m comfortable with the authors doing what they think is best in this instance: given that the derivations are, at least, technically correct, this is a stylistic matter.

There are a few problems that remain, however: a few of these are responses to the authors’ questions to me; others mainly involve continued logical inconsistencies and conceptual confusion about the nature of their system model. All of these are fairly easy to address, with only relatively small edits required in each case.

My comments are itemized using the labels from the authors’ responses. 3.8(a)

We are glad that the reviewer finds our changes agreeable and have made further edits in the hope of rendering the manuscript acceptable.

3.8(a) The paper now includes R2 estimates, but a few issues remain:

1. Citing values from other studies is not particularly relevant. We can’t compare experimentalists and data in this way: one group’s experimental results might simply be better than another’s. At most, such citations indicate that it’s possible to get good experimental results. It has no bearing on whether the results of this particular paper are accurate.

2. What does the range mean? We don’t have a sense of how many of the estimates were near which end of the range. Why not show a box plot of the R? values? At least give us the mean/median and some mention of variance or confidence intervals.

3. Most importantly, the R2 is for the simultaneous estimate of both parameters. This misses the main point of my original comment, which was about each parameter individually. That is, my main question was about whether or not both the proportional and derivative controllers are needed to capture your data. So, the question is: how much variance do bd and bp each on their own capture? The

fact remains that bd fits the data very well at both walking speeds, whereas bp does not.

Our goal here was to show that the linear regressions used here generally have R^2 values that are considered good to very good, in response to the reviewer's request to discuss the goodness of fit. We agree that the added text lacked detail and have edited to clarify. We replaced the ranges with estimates of the mean and standard deviation. To determine how much variance bp and bd each capture on their own, we analyzed the data set by fitting two different types of model, with CoM position or velocity as a single predictor for foot placement. We find that the model with position fits the data substantially better than the model with only velocity, but the combined model with two predictors is substantially better again, and for all subjects and speeds, both predictors are significant ($p<0.0001$ in all cases). We added the following text to the discussion:

> Here we found adjusted R2 values around 0.754 ± 0.0966 (mean ± standard deviation) for normal walking to a metronome at 110 beats per minute, and 0.666 ± 0.132 for slow walking at 80 beats per minute. Between the two predictors, the CoM position at midstance tends to have more explanatory power for the foot placement than the velocity. When using either variable as a single predictor, the adjusted R2 values for normal walking drop to 0.497 ± 0.158 for the model with position as single predictor and 0.246 ± 0.129 for velocity. However, the velocity still has substantial explanatory power and is significant as a predictor ($p < 0.0001$ for all subjects). This indicates that even though bd is not well predicted by the model, and the estimates tend to vary more between subjects, the CoM velocity is important for the neural controller to adjust foot placement location with the goal of maintaining upright stability.

3.S.3 In responding to my comment on the four-dimensional nature of the system's phase space (or state space), the authors have added a paragraph starting with "|t]he only difference between the two versions of the system describing progressive walking in the anterior-posterior direction and alternating walking in the medial-lateral direction is the addition of the alternating sign of the offset;" and concluding with "[t]he dynamics of the actual system are, of course, three-dimensional."

Unfortunately, this paragraph suggests that the authors may still not have clarity about the actual nature of the system they're looking at. First, there is only one system with one center of mass, not "two versions" of the system. Each alternating step and each progressive step is taken at the same time. Second, when they say that the system is "three dimensional", they seem to be confusing the dimension of physical space the walker moves in, which is what they mean by

three, with the dimension of the walker's state space, which is four. The latter is what my comment was referring to. The authors have glossed over this aspect of my comment. On top of that, in saying that the walker moves in three physical space dimensions, the authors contradict their other statements elsewhere in this document (3.8.26) that it moves in a plane (which is true-to leading order, the CoM moves in the horizontal plane).

The core of the misunderstanding here seems to be that the word "system" has multiple meanings, and we sometimes neglected to specify which one we are currently using. For most of the paper, we are discussing dynamical systems, which formally is a mapping from the cartesian product of a state space and a time space into the state space. In our case, the state space consists of two variables, *x* and *v*, and the dynamical system arises from the differential equations given in Equation 1 of the paper. These equations are designed to describe, with several approximations and simplifications, some aspects of physical reality, specifically how a human walks through space. Physical space is three-dimensional, but the dynamical system analyzed in the paper only describes movement in one of these three dimensions. With minor changes in the equations, this can be either the medial-lateral or the anterior-posterior direction. We edited the text to further clarify this.

> We also note that analyzing movement in the anterior-posterior and medial-lateral directions as separate, one-dimensional dynamical systems is possible due to the simplifying assumptions made here, specifically the linearization, lack of swing leg dynamics and constant step time. The actual human walking through physical space is, of course, three dimensional, and our dynamical system define by Equation 1 describes one of the three dimensions of physical space.

3.S.3 I understand that the triangle plots don't require the parameters to be the same. That was precisely my point: the different directions do not have to lose stability together. It's possible, for example, for the progressive direction to be stable and the alternating direction to be unstable. Yet, the manuscript says, in referring to Kuo's 3D walker (lines 731-732 of the revised manuscript):

"Our model fails to predict this difference between frontal and sagittal plane stability." This is false. Just as with Kuo's model, your model can lose stability laterally and not in the sagittal direction if parameters are chosen to permit it. See also my responses to 3.8.4 and 3.8.27.

We agree with the reviewer that it is possible to lose upright stability in one direction while the other direction remains stable, this is not what we were discussing in the cited

passage. We believe that this another misunderstanding, at the heart of which are different meanings of the term “stability”. In the cited passage, we use stability as a property of a point or trajectory of a system. As clarified in Section 2.3, it indicates asymptotic stability of a periodic orbit. The reviewer, on the other hand, refers to “loss of stability” as an event, i.e. something that happens at a specific point in time.

To address the remaining points brought up by the reviewer here, we added the following text after the cited passage.

> Our model fails to predict this difference between frontal and sagittal plane stability, because the criterion for the stability of a walking system, stated in Result 2, is the same for the progressive and alternating version of the walking system. This implies that the reasons for the difference between frontal and sagittal plane stability reported by Kuo et al. are not based on alternating vs. progressive stepping, but rather due to features not currently included in our model, such as the foot being much longer than it is wide, or the neural control parameters being different for the two directions.

3.S.4 This is a continuation of the issue discussed in my response to 3.8.3. The problem is the authors keep saying their stability results are different from those found with Kuo’s 3D walker. But they also say: “Specifically, we do not assume that the values would be identical for the two directions of a 2d system”. Good! Perhaps what they’re trying to say is that the stability analysis is identical in both directions, which is true—but the actual stability doesn’t have to be the same because the parameters don’t have to be the same. Thus, when you say that this is different than Kuo’s model you are being logically inconsistent and, therefore, incorrect. Your model allows each direction to be stable or unstable independently of the other, depending on the parameter values chosen, so you can make it behave the same as Kuo’s model. In other words, the model doesn’t just have two parameters, {bp, ba}, it has four, which might be written {b", ba, br .be \, where the superscripts A and P indicate the alternating and progressive directions, respectively.

We hope that we clarified some of these issues with our response to 3.S.3 above. We would also point out that comparisons with Kuo’s results must, more or less, necessarily fall short, since the mechanics are different. The mechanics of Kuo’s model are substantially different between the sagittal and the frontal plane, leading to passively stable modes in the sagittal, but not the frontal plane, which has to be stabilized by active control.In our model, the mechanics are identical in the two planes, with no passively stable modes, so both directions have to be stabilized actively.

3.S.17 The authors are "confused by my confusion", so let me be more direct: it is absurd to say that MoS defines "when people will have to take a step" when applying it to gait, during which people always have to take steps to keep walking.

We agree with the reviewer, and point this out ourselves in the fourth paragraph of the introduction.

The authors justify this description by applying the MoS concept to the task of standing, which is a poor paradigm to use for walking.

Again, we wholeheartedly agree, this is pretty much exactly the point we are trying to get across.

Yet, the solution to the problem is right there, when they quote Hof: "the MoS is the minimum distance from the XCoM to the boundaries of the BoS." That's it! That's what they should say the MoS is because that's its definition.

And we do say that!

When walking, if the XCoM exceeds the BoS, the mass center will overshoot the base of support (defined by the current stance foot) before the next step occurs.

We also do not disagree with this statement.

3.S.18 The authors remain attached to the notion that stability theory is "mathematical extremism." On lines 497-504 of revised manuscript, they say [underlining mine}:

> **In dynamical systems theory, asymptotic stability is a well-defined property of a fixed point or periodic orbit of a system [25]. In typical mathematical extremism, this defines a "substantial change" as any change at all following an infinitesimally small perturbation after an infinitely long time. While extreme, this definition and associ- ated methods from dynamical systems theory have also been widely applied to the analysis of walking movements, mostly in the form of measures that quantify how fast a system relaxes back towards the stable state after a perturbation, e.g. Lyapunov exponents [26], Floquet multipliers [27].**

I'd say that that the mathematical description of stability presented by the authors may sound "extreme", but that's only because they have done such a poor job of it. Perhaps they are deploying a straw man argument, in an attempt to criticize work they feel is overly mathematical. Or, they might simply be attempting to be

humorously descriptive. Maybe it is just unclear writing. I certainly can't divine their motivations. But, intentionally or not, they obscure the relatively simple meaning of stability. If the authors really want to reach non-experts (which they claim they want to do, and is why they want to include the elementary math now moved to the supplemental material), it is a disservice to that community to confuse them with an inadequate description of such an important concept.

Stability can be understood without boggling people's minds. For example, it took me thirty seconds to find this simple, nonmathematical description on Wikipedia:

> **Stability theory addresses the following questions: Will a nearby orbit indefinitely stay close to a given orbit? Will it converge to the given orbit? In the former case, the orbit is called stable; in the latter case, it is called asymptotically stable and the given orbit is said to be attracting.**

There's this more authoritative plain-English description on Scholarpedia:

> **The stability of an orbit of a dynamical system characterizes whether nearby (i.e., perturbed) orbits will remain in a neighborhood of that orbit or be repelled away from it. Asymptotic stability additionally characterizes attraction of nearby orbits to this orbit in the long- time limit.**

Notice neither of these refer to "infinitely long times" or "infinitesimally small perturbations". They are technically correct but accessible to non-experts.

This need not be difficult. Frankly, another option for the authors would be to simply remove most of the underlined portions of the above-quoted text from the manuscript. The remaining paragraph would still be perfectly serviceable.

Our motivation for the term "mathematical extremism" was a bit of self-deprecating humor. We appear to have deprecated others along with ourselves, including the reviewer, which was not our intention, so we decided to remove that term.

We would like to point out that in the two definitions of stability cited by the reviewer, the "infinitely long times" and "infinitesimally small perturbations" are hidden rather than absent. Because "indefinitely" is pretty much another word for "infinitely long", and by "will remain in a neighborhood" the authors most certainly don't mean "for at least half an hour". The history of what we now call calculus is full of conceptual problems since its introduction by Newton (and others) in the 1600s, using "infinitesimals" that he never was able to formally define. A formal solution was developed by Weierstrass in the 1800s using sequences and limits, and later this formalism was reconciled with Newton's concepts by Robinson, who was able to formally construct infinitesimals within

the framework of the standard approach (Non-standard Analysis. Abraham Robinson. Princeton University Press, 1974). The Weierstrass framework is still used in the definition of asymptotic stability of a fixed point in a dynamical system today, and it is present as well in the definitions cited by the reviewer, in terms like “converge” or “attraction”, whose formal definitions rely on sequences and limits. Our formulation in the manuscript certainly puts the focus on the infinites and infinitesimals, rather than hiding them, and that is just our intention. But rather than trying to boggle the reader’s mind, our goal is to point out that formal definitions of the term “stability” require such extreme concepts – in contrast to actual walking, where perturbations are never infinitesimally small, or recovery times infinitely long. Both viewpoints are relevant, but it is critical to clarify which viewpoint one is taking and which meaning of terms such as “stability” one is using in any given conversation, to avoid misunderstandings, as discussed in the “What does stability mean” section in the Discussion.

3.S.26 The authors state: “In a horizontal plane, the vertical dimension is, by definition, constant. We do not understand which aspect of that is only approximal and respectfully ask the reviewer to elaborate.” The authors join all other researchers in referring to their model as an inverted pendulum. However, the pendulum is a nonlinear mechanical system. The mass at the tip of an inverted pendulum can obviously change its height, as is obvious if one looks at a picture. It is only for small angular deflections (or, equivalently, small displacements) that the tip mass can be said to approximately not change its height and to stay approximately in a horizontal plane. That is, the inverted pendulum model the authors (and Hof, and others) use is a linearized inverted pendulum, obtained by Taylor series expansion about the vertical position. The vertical position is only constant to leading order in the displacements.

We thank the reviewer for the clarification and assure them that we are aware of the difference between the linearized and the non-linearized inverted pendulum. We stated in the manuscript that we use the linearized version, following Morasso and Kajita. We have made this more clear by editing the text as follows

> We linearize the equations of motion [15], which effectively movement in the vertical direction and constrains the CoM to move in a horizontal plane parallel to the ground. This linearized system, which we will simply refer to as inverted pendulum from now on, is determined by the equations of motion ~~After linearization [15], the equations of motion describing how the body accelerates relative to the contact point are given by~~

3.S.27 I have already discussed this multiple times. The authors state: “The contrast between our work and Kuo’s lies in the fact that our model predicts that

stability is the same in the frontal and the sagittal plane, while Kuo, and others, find that it is not." As stated in my responses to 3.8.3 and 3.8.4, this is false. Your model is perfectly capable of having different sagittal and frontal plane stability. All you have to do is set the parameters differently in the two directions so that this occurs.

We agree with the reviewer that our model does not *predict an identity* between the two directions, as we state in the previous response to the reviewer document. Rather, it *fails to predict a difference*, as we write in the manuscript. This is an important logical difference and we apologize for having mixed this up.
